# Ringed Seal Search for Global Optimization via a Sensitive Search Model

**DOI:** 10.1371/journal.pone.0144371

**Published:** 2016-01-20

**Authors:** Younes Saadi, Iwan Tri Riyadi Yanto, Tutut Herawan, Vimala Balakrishnan, Haruna Chiroma, Anhar Risnumawan

**Affiliations:** 1 Department of Information Systems, University of Malaya, 50603 Pantai Valley, Kuala Lumpur, Malaysia; 2 Department of Computer Science, University of Ahmad Dahlan, Jalan Kapas n 9, Yogyakarta, 55165, Indonesia; 3 Department of Computer Science, Federal College of Education, (Technical), Gombe, Nigeria; Jiangnan University, CHINA

## Abstract

The efficiency of a metaheuristic algorithm for global optimization is based on its ability to search and find the global optimum. However, a good search often requires to be balanced between exploration and exploitation of the search space. In this paper, a new metaheuristic algorithm called Ringed Seal Search (RSS) is introduced. It is inspired by the natural behavior of the seal pup. This algorithm mimics the seal pup movement behavior and its ability to search and choose the best lair to escape predators. The scenario starts once the seal mother gives birth to a new pup in a birthing lair that is constructed for this purpose. The seal pup strategy consists of searching and selecting the best lair by performing a random walk to find a new lair. Affected by the sensitive nature of seals against external noise emitted by predators, the random walk of the seal pup takes two different search states, normal state and urgent state. In the normal state, the pup performs an intensive search between closely adjacent lairs; this movement is modeled via a Brownian walk. In an urgent state, the pup leaves the proximity area and performs an extensive search to find a new lair from sparse targets; this movement is modeled via a Levy walk. The switch between these two states is realized by the random noise emitted by predators. The algorithm keeps switching between normal and urgent states until the global optimum is reached. Tests and validations were performed using fifteen benchmark test functions to compare the performance of RSS with other baseline algorithms. The results show that RSS is more efficient than Genetic Algorithm, Particles Swarm Optimization and Cuckoo Search in terms of convergence rate to the global optimum. The RSS shows an improvement in terms of balance between exploration (extensive) and exploitation (intensive) of the search space. The RSS can efficiently mimic seal pups behavior to find best lair and provide a new algorithm to be used in global optimization problems.

## Introduction

In the last recent years, several metaheuristic algorithms have been introduced. The significance of using such approaches to solve optimization problems justifies their popularity. The metaheuristic optimization algorithms-based population is one of the useful models where its principle usually starts with an initial set of variables, and proceeds to a specific process to obtain the global minimum or maximum of the objective function. Genetic Algorithm (GA) is considered as one of the most popular approaches [1]. It uses operators inspired by natural genetic variation and natural selection [1–4]. Particles Swarm Optimization (PSO) was inspired by the fish and bird swarm intelligence [2]; on the other hand, Firefly Algorithm (FA) was inspired by the flashing pattern of tropical fireflies [5–9]. The Cuckoo Search (CS) was inspired by the brood intelligent behaviour of some cuckoo species. Its strategy consists of laying its eggs in other cuckoos’ nests [10,11]. A very huge number of researches have introduced the use of metaheuristics approaches to resolve optimization problems, particularly NP-hard problems, such as Travelling Salesman Problem (TSP) and Minimum Spanning Tree Problem (MSTP) [3,6–8,12–14]. The main advantage of metaheuristics algorithms is the ability to keep good performance in dynamic changes [1]. This power of robustness comes from the fact that they imitate natural phenomena that have existed and evolved on the earth since millions of years. Particularly, a metaheuristic algorithm is considered as robust only if it fulfils two requirements: intensification and diversification [6,15]. Intensification consists of exploring the local search area to find the best quality of solutions whereas diversification consists of ensuring that the algorithm is able to be efficient in covering the entire search domain. Therefore, the ability of a metaheuristic algorithm to find the global optima is in correlation with its capability to find an optimal balance between the intensification (exploitation) and the diversification (exploration) of the search space.

Various studies showed that CS outperformed GA, PSO and other conventional algorithms for global optimization problems [10]. This is partially due to the fact that CS which is based on Levy flights shows an optimal balance between exploitation and exploration, which has a major impact on the algorithm efficiency [16]. On the other hand, GA, PSO and CS are potentially dominating the global optimization algorithms that are used explicitly or implicitly for many applications in science and technology, and they have been used to compare many new developed algorithms [10,11,17–21]. However, they show a weakness in terms of the balance between the exploitation and exploration during search for new solutions [22,23]. For example, in multi-objective problems the search is not intensified on the visited regions effectively and oftentimes it usually shows a precocious convergence and lack of diversification. In order to treat this problem, various approaches have been proposed in the literature [9,24–34]. In most of the introduced approaches, extensive and intensive search are adjusted using the parameters setting, however this has an impact on the search abilities of the algorithms to deal with multi-objective problems [29]. Another popular technique used particularly in evolutionary algorithms consists of starting an intensive search, and then gradually exploring other locations until all the search space is covered [26,30]. However, such techniques make solving of multi-objective problems difficult especially in cases where the problem holds many optima.

Many other proposed approaches can be found in [9,23,30,32,35]. Another significant approach used in metaheuristics for exploring the search space is randomization [2,6,16,22,36]. Randomization to balance between exploration and exploitation can also be used to jump the local optimum, which gives the opportunity to explore the search space efficiently. On the other hand, randomization can be used to perform an intensive search at the local region around the current best solution. Randomization can also be found in some approaches combined with stochastic rules [37–40]. Some other approaches are based on complex methods such as Monte Carlo simulations [41]. They can also be more detailed such as the computational model proposed by Nurzaman [42–46]. It is shown that for low target density regions, Levy walk performs well. However, Brownian walk performs better when the targets are abundant [47]. Exploitation consists of the search for knowledge at the search space, and using the found solution for defining the new search moves at the local area where the local optimum may be located. However, sometimes the local optimal cannot be the global optimum. It is shown that too much of exploitation increases the possibility to be trapped in a local optimum [16]. On the other hand, a strong exploration increases the possibility to find the global optimum but with less efficiency [23]. The first and only theoretical basis found in the literature for the optimal exploitation and exploration for multi-objective problems was introduced by Yang *et al* [16]. The study shows the ratio of search times interpreted by efforts of exploration and exploitation stages, it also provides some efforts done on the global exploration which is in correlation with the local exploration. Thus, there is a need for a mechanism to balance the right value of exploration and the right dose of exploitation. Although big efforts have been done on search approaches, there is no specific guideline for balancing exploitation and exploration, resulting in each heuristic algorithm to have a different method of exploration and exploitation.

In this paper, a novel nature-inspired metaheuristic algorithm, known as the Ringed Seal Search (RSS) is proposed for solving global optimization problems. The RSS is based on the search behavior of seal pups to find best lair to escape predators. The sensitive nature of seal pups against external noise has a big impact on their search, especially against the noise produced by polar bear movements. Seal pup movement is based on two search states, normal state and urgent state. In normal state, the pup moves between closely adjacent lairs (intensive search). In urgent mode, at low temperatures, during polar bear movement, ice transmits noise very well; the pup leaves the proximity area far away (extensive search) to find a new lair from sparse targets. Some approaches focus on enhancing the conventional metaheuristic algorithms by merging some techniques for balancing the exploration and the exploitation dose. Examples include incorporating Levy flights for revisiting targets and using intermittent search strategy for non-revisiting targets [48,49]. In contrast, the proposed RSS algorithm introduces the sensitive search model inspired from seal movement. The sensitive search model incorporates Levy walk and Brownian walk, known for their potential capacity for exploration and exploitation, respectively [43,46,47]. The sensitive search model divides the search into two states: normal and urgent. At each state, the seal pup shows a different movement behavior. By default, at the beginning is the normal state (exploitation) where the pup moves at the multi-chambered lair, the algorithm changes the state of the search into urgent state in case of emitted noise, and thus the pup moves far away to find a safe lair. The noise is characterized by a uniformly distributed pseudorandom integer that is transmitted randomly. The algorithm keeps switching between normal and urgent states until the optimum is reached. As a result, the sensitive search model can ultimately improve the balance between exploitation and exploration of the RSS, likewise it also maintains the search behavior of seal pups observed in nature. To explain the proficiency and robustness of RSS, it was compared to GA, PSO and CS. The comparison investigates different standard benchmark functions that are usually used to test global optimization algorithms [10,17–21]. Experimental results show that the proposed RSS algorithm is faster in finding the global optima over its homologs, as a result of balance between exploitation and exploration.

The rest of the paper is organized as follows. First, the RSS behavior is presented. After then a description of the seal’s sensitive search model is introduced. In accordance with that, the description the proposed algorithm is presented in detail. After then a discussion of the experimental results is introduced. A case study about Field-Programmable Gate Array (FPGA) using RSS is introduced. The contribution of this work is highlighted, followed by the significance of the proposed algorithm. Finally, the conclusions of this work are formulated.

## Ringed Seal Search Behaviour

Optimization is a substantial challenge for organisms, where escaping predators searching for habitats and foraging defines their behaviour. The mechanism used by organisms to search optimally to get best habitats is developed through hundreds of years in nature. In this paper, the focus is on ringed seal which is a semi aquatic animal, not only because of its extraordinary ability to stay and dive underwater for a long time, but also because of its amazing behaviour used to resist natural fluctuations. This behaviour is developed since thousands of years, making the seal to be adaptable to unexpected and difficult conditions. As all semi aquatic animals, underwater activities of diving for seals are constrained by the need for surface gas exchange. The seal breeding also requires a suitable environment to guarantee the reproduction of new generations [50].

During autumn and winter in the Canadian arctic, the ice starts freezing over, so the seals create breathing holes and snow covered lairs. Between March and May, ringed seals give birth to pups in snow-covered lairs connected to the ocean. These lairs provide a thermal protection against cold air temperatures and high wind chill, and afford at least some protection from predators such as bears [51–53]. A seal could have a complex of lairs at one specific area [54–56], which can be used for many functions: breeding and birthing of young pups and resting. Lairs are maintained until the end of the breeding season in spring, approximately six weeks after pupping, or until snow melt causes structural collapse [51]. In nature, two different types of lairs were observed [57]. Generally, the famous type in both coastal and offshore habitats is haul-out lairs, which is characterized by a single- chambered room and has a round design. Another different type of lairs found is called the birth lair. A birth lair can be characterized by the existence of placental remains, hair and also by extensive tunnels created by pups. The seal pup strategy consists of searching the best lair to avoid predators. The young pup moves between lairs within her complex of lairs. If a lair is attacked, destroyed or its quality not good, pups are able to change the location between lairs structures [56,57]. The search movement of the seal is sensitive to external noise emitted by predators such as polar bears. In case of noises, the pup leaves the proximity far away. However, in normal situation where there is no external noise; the pup keeps browsing the proximity (the multi-chambered lair) searching for best location. Basically, the quality of the habitat depends on the structure of the lairs, therefore during the breeding season male ringed seal emits a strong gasoline smell which may indicate the location of the lairs [57,58]. Wounds on both males and females represent another smell index that can mark territories. This makes seals very vulnerable and unsafe and could be targeted by bears. A polar bear can locate seal lairs using the smell index [52]. Its strategy consists of sniffing the ice surface with self-possession searching for a seal meal; if a smell is detected, the polar bear will run and jump on the snow over the hole to collapse the lair and block the exit. The bear can then catch the mother and the pup together. The ringed seal strategy used to search and choose the best lair can be associated with the objective problem to be used to balance between the exploitation and the exploration of the search. The proposed approach is based on the randomized noise emitted by predators to combine different search patterns for the seal to design a new algorithm for global optimization problems. The seal sensitive search model is described in the following section.

## Description of the Seal’s Sensitive Search Model

Generally, in nature a lot of organisms perform random search during foraging and searching for resources such as food and water. Several recent studies show that many animals perform random search based on statistical procedures [42,59–64]. One of the random walk techniques that have got much interest is the Levy walk, which is characterized by a heavy tailed step length distribution. On the other hand, some new introduced search techniques [47,65] show that Levy walk performs better for search with sparse targets. In contrast, Brownian walk is more efficient where the step lengths are not heavy tailed. The aim of this section is to describe the search behavior of the seal pup during normal and urgent state. Particularly, the movement of seal pup is characterized by a high sensitivity to external noise as shown in the figure below.

**Fig 1:**
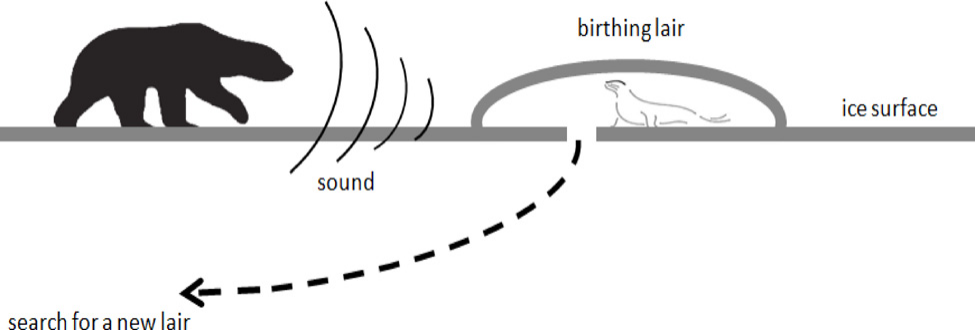
Seal’s movement when leaving the lair (urgent state).

Fig 1 shows a seal pup inside a birthing lair, on the other side a bear in movement on the ice surface. In case of an urgent state, the seal pup strategy consists of two options, keep silent and wait for unknown destiny, or jump inside the sea through the hole to find another lair to escape the predator.

Recent researches showed that some noise-based strategies, namely biological fluctuation has an effect on the life sciences [66]. This strategy also exists in many varieties of bacteria, where its role consists of providing an adaptation to environment changes. Stimulated by this natural phenomenon, several models have been introduced to explain the biological fluctuation [42,66,67]. The movement of seal is also characterized by sensitive reaction to external noise. The search of the seal is therefore designed to have two different patterns, normal search (normal state) where there is no noise or urgent search (urgent state) in case of noise.

**Fig 2:**
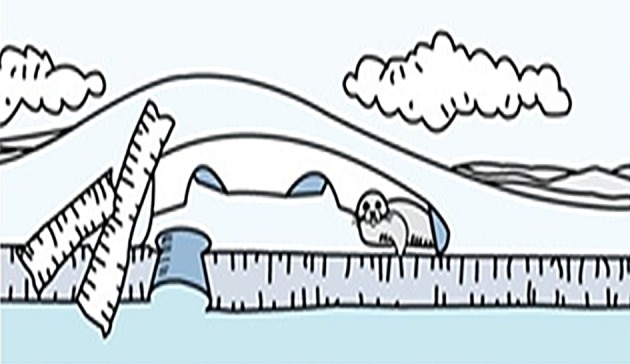
Seal inside a multi-chambered lair during a normal state, designed by Robert Barnes, UNEP/GRID-Arendal [68].

For the urgent search state, the seal pup leaves its own lair and performs a long step lengths using a Levy walk as shown in (Fig 1). The purpose of this long step search pattern is to escape the external noise threat emitted by the predator and explore if other lairs could be safer. In terms of global optimization point of view, this could be interpreted as an exploration of the search space. For the normal search state, the seal exploits the local area searching for a better location as shown in (Fig 2). In contrast to the urgent state, in normal state the seal is not threatened by an external noise and that is an enough reason to keep exploiting the proximity of the current lair.

Fig 2 shows a seal pup inside a multi-chambered lair. In the absence of external noise, the seal prefers to exploit the local area (the chambers of the lair). This represents a normal search state when the seal pup performs a Brownian walk with a non-heavy tailed step length that can be interpreted as an intensive search at the proximity (exploitation). In nature, one mother seal can have a complex structure of lairs at one place.

### Why Levy Walk and Brownian Walk?

In mathematics, the definition of a random walk is introduced as a formalization of a path that is constructed using a series of successive random steps. Levy walk is considered as one of the random walk techniques that are potentially used to model animal movement. It is characterized by a specific step size (random number), where the length between two consecutive portions of direction is calculated from a probability distribution with an inverse power-law tail described as below [65,69,70].

$$\text{Levy}∼u=t^{-\lambda },$$ (1)

where 1 < *λ* < 3 and *t* is the flight length. In fact, the generation of random numbers with Levy walk particularly consists of two main steps: the selection of a random direction and the calculation of step size which is in conformity with Levy distribution. Selecting a random direction is calculated through a uniform distribution. In case where *λ* ≥ 3, the distribution will not be in a heavy tail and the total sums of the lengths converge to a Gaussian distribution. Levy walk is characterized by an anomalous diffusion, where the mean squared displacement increases faster linearly with time. However, Brownian walk is characterized with a normal diffusion where the mean squared displacement increases linearly.

In [43], it is shown that animals perform Levy walk patterns during search for resources that are distributed in different patches. For this, animals use two modes, intensive mode to concentrate on the search inside the patch (exploitation), and extensive mode to move from one patch to another (exploration). In [44,46,64], it was shown that animals route is quite similar to Levy walk. However, some models demonstrated that when prey resources are abundant, Brownian walk is performed by animals whereas when preys are distributed into different patches Levy walk is performed [64]. In [42] a model of Levy and Brownian is presented, showing how Escherichia-Coli switches from Levy to Brownian mode based on target densities. Implicitly, the main question is what mechanism animals use to switch from one mode to another. As explained above, the seal search used to find other lairs (exploration) is in correlation with the presence of the external noise. However, in the opposite case where there is no external noise, the seal stays at the same lair and keeps exploiting the multi-chambered lairs. Based on this approach, the seal search can be divided into two states: normal and urgent. In each state, the individual (seal pup) exhibits a specific walk pattern (Levy or Brownian).

### The Formal Definition of the Sensitive Search Model

**Fig 3:**
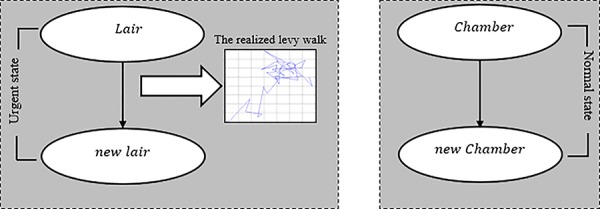
A model of Seal Search during urgent state (a), and normal state (b).

The movement of the seal pup inside its multi-chambered lair or during search for new lairs can be described as a series of events. Formally, let (Ω,*β*,∂,*ρ*) be a search space that contains *β* predator and ∂ seal pup. In the interpretation, (Ω,*ρ*) is the state of the search space. If the current state of the search space *ρ* is *ω* where *ω* = 1 (*ω* represents the external noise), then ∂ is informed that Ω contains *β*, which is a predator emitting a noise *ω* during movement. Given *E* event in Ω, a state (Ω,*ρ*) is called urgent state if Ω includes *β* and ∂ members of the event at the search space that contains the noise *ω*. Let *A* be an event where (Ω,*β*,∂,*ρ*) is the search space. If the current state of the search space *ρ* is *ω* where *ω* = 0, then ∂ is not informed that Ω contains *β*, then (Ω,*ρ*) is considered as a normal state. In urgent state ∂ performs a Levy walk, however in the opposite case (normal state) ∂ performs a Brownian walk. Considering this description of the search space, the movement of the seal pup from a lair to another (urgent state) can be described as shown in Fig 3(A).

From Fig 3(B), moving from a lair to a new lair requires a specific search pattern. During the generation of new solutions (new lairs) *x*^(*g*+1)^ for, say, a seal *i*, a new lair is found:

$$x_{i}^{g+1}=x_{i}^{g}+\alpha ⊕\Delta x,$$ (2)

where *α* is the step size which is related to the search pattern, during normal or urgent state.

$$\Delta x=\{\begin{array}{l} \;Levy\left(\lambda \right),\;\omega =1\\ Brownian\left(\lambda \right),\;\omega =0 \end{array},$$ (3)

where *ω* is considered as a pseudo-random integer from a uniform discrete distribution. In case of Levy walk, the random walk is characterized by a step size calculated from a probability distribution with an inverse power-law tail as shown in Eq 1. In case of Brownian walk, the search for a new chamber inside the structure of a multi-chambered lair as shown in Fig 3(B), the search is characterized by a step size described as below.

S=(k*randn(d,Ndots)). (4)

where *k* is the standard deviation of the normal distribution for diffusion rate coefficient, *d* is the dimensions of the problem and *Ndots* represents the number of particles of the Brownian in the search space.

## The Algorithm

Ringed Seal Search (RSS) is particularly based on seal pup search for best lairs to escape predators. Everytime a new lair with a good quality is found, the pup will move into it. At the end, the lair (habitat) with the best fitness (quality) will be the term that RSS is going to optimize. The RSS scenario is based on the following representations:

1. Each female seal gives birth to one pup at a time in a specific habitat chosen randomly.
2. The seal pup moves randomly inside its ecosystem to find a good lair to escape predators.
3. The movement of the seal pup can take two states: Normal where the search is intensive using a Brownian walk or Urgent where the search is extensive using Levy walk.
4. If *L*^*best*,*k*^ the best seen lair from the current *K* of the existing lairs *L*^*best*,*k*^ is better than *L*^*best*,*k*−1^ the best of the previous iteration in term of fitness value, *L*^*best*^ is updated to be *L*^*best*,*k*^, otherwise *L*^*best*^ remains with no update.
5. Gradually, worse lairs will be abandoned and seals continue moving to other lairs (or chambers) (convergence to good solutions).

**Fig 4:**
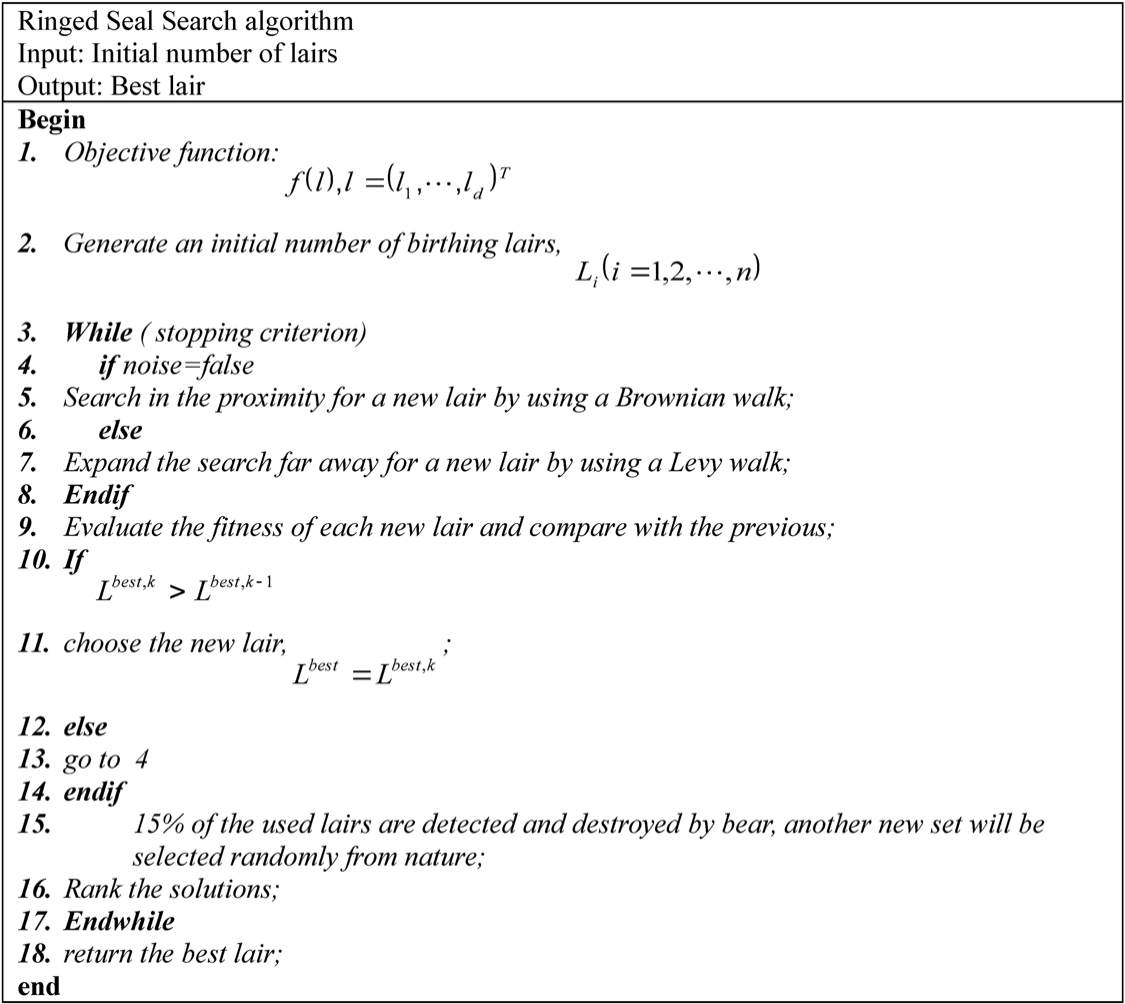
The Ringed Seal Search algorithm.

The number of lairs is fixed where the mortality rate of seals is interpreted by the rate of lairs destruction which is equal to 15% [58]. The complete algorithm is divided into three main parts. The first part corresponds to the initialization stage, while the remaining two stages represent the search for new solutions (lairs) and abandonment of worse lairs, respectively. All the optimization processes consists of a vector of values *L*_*i*_ (*i* = 1,2,⋯,*n*) representing the initial solution. The overall process of optimization is described in Fig 4.

Fig 4 shows the main skeleton of RSS. The algorithm starts with an initial number of birthing lairs *n*. In this algorithm we assume that the lairs are multi-chambered. The pups move in the search space to get new lairs with better quality. For each new found lair, the fitness is evaluated based on the seal’s decision to move, if the new lair is better than previous. After ranking the lairs, the RSS selects randomly 15% from the search space and replaces the worse lairs. At the end, according to the stop criterion, the RSS returns the best lair. The main steps of Fig 4 are described in details as below:

### Generating Initial Lairs

Solving an optimization problem always starts with initial values. For that it is necessary that these initial values be formed as an array. Here in RSS algorithm the values represent the lair in which the seal pup is living. The lair is defined as below:

$$L_{i},i=\left(1,2,\cdots ,n\right).$$ (5)

The lairs are distributed randomly, and each lair *l* contains many chambers *m*. For example, for a lair *i*, it is an array of [1 × *m*], representing current existing lair *l* of a habitat.

L=[1×m]. (6)

***Such values are randomly and uniformly distributed at the search space between the pre-defined lower bound*** *Lb*_*j*_ ***and upper bound*** *Ub*_*j*_**, *as illustrated in the following expression*:**

$$L_{i}=Lb_{}+\left(Ub-Lb\right).rand\left(size\left(Lb\right)\right)$$ (7)

i=(1,2,⋯,n)

where *i* represents the number of the lair and *n* indicates the number of the initialized lairs.

### Seal’s Search for Lairs

During each iteration, the pup performs a movement and selects a lair randomly (a new solution). The movement can be in two different patterns: in normal state using Brownian (intensive) or in an urgent state using Levy walk (extensive). For each mode there is a specific type of random walk, where the steps are determined in terms of the step length, with a specific probability distribution with the search direction being random. The main operators of search are described as below:

#### Random Noise

In order to simulate the random external noise emitted by predators, the proposed algorithm generates a uniformly distributed pseudorandom integer to model the noise *ω*. The noise *ω* takes two values: *ω* = 0 and *ω* = 1. If *ω* = 0, the search space state (Ω,*ρ*) will be in a normal state, and an intensive search (exploitation) will be performed by the seal pup at the proximity of the multi-chambered lair. By contrast if *ω* = 1, the search space (Ω,*ρ*) state will be in a urgent state, as a result the seal performs an extensive search to find a new lair (exploration).

#### Normal State

In the normal state, the random noise value *ω* = 0, then the seal ∂ experiment a normal behavior and search. Such state is characterized by a random movement at the proximity of the multi-chambered lair. Therefore, the movement is modeled via a Brownian walk as a non-heavy tailed step length that can be interpreted as an intensive search (exploitation).

#### Urgent State

In the urgent state, the seal is threatened by the external noise emitted. As a result, the search space (Ω,*ρ*) takes an urgent state affected by the external noise emitted *ω* = 1. This state is characterized by an extensive walk in the search space. It is modeled via a Levy walk which is known by a heavy tailed step length distribution which is suitable for search in case of sparse targets. The nature of the urgent state case stimulates the seal to move outside the lair, and tries to get another solution to escape the predator’s threat.

### Best Lair Updating

Despite the fact that this updating process is not a part of the Sensitive Search Model, it is used to simply select and store the best so far solution (lair) found. In order to update the best lair *L*^*best*^ found so far, the best seen lair from the current *K* of the existing lairs *L*^*best*,*k*^ is compared to the best lair of the previous iteration *L*^*best*,*k*−1^. If *L*^*best*,*k*^ is better than *L*^*best*,*k*−1^ according to its fitness value, *L*^*best*^ is updated to be *L*^*best*,*k*^, otherwise *L*^*best*^ remains with no update. Thus *L*^*best*^ memorizes the best historical lairs found so far.

### Abandoning Worse Lairs

After all the seals have moved to new lairs, certain lairs with high smell index will be detected and destroyed by the bear. The percentage of destruction of lairs (mortality rate of seals) is set to 15% by default, the same rate found in nature [42], and it can be modified according to the nature of optimization problem. These abandoned lairs will not be suitable to host pups again and will be abandoned definitively. The rest of the lairs will host pups until the pups decide to leave due to one of the reasons below:

1. The snow covered the lair has melted.
2. A predator attacks the lair, so the seal escapes the area.

Another interesting feature for seal pups is that one lair can be used communally by different seal pups [58], something that occurs rarely in nature.

### Convergence to Optimal Lairs

After certain iterations, all the seal pups move to a new lair (new solutions), which is better than the previous locations. These newfound lairs will provide better protection to the pups to avoid the predator’s threat. As a consequence, there will be less killed seal pups by predators, which can ensure the reproduction of new generations. The fast convergence to the optimal locations (lairs) ends the RSS algorithm quickly.

**Fig 5:**
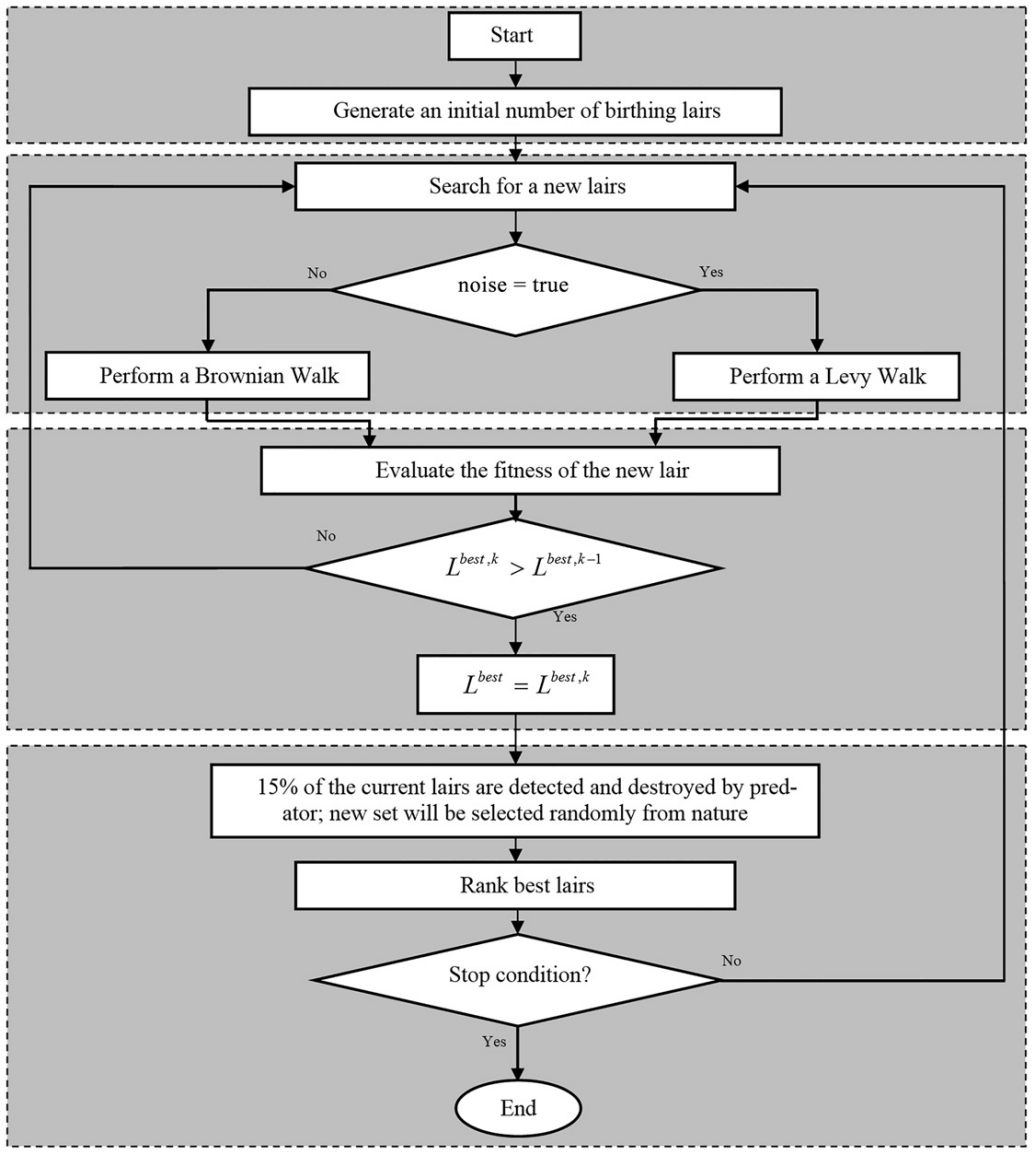
Data flow in the RSS algorithm.

Fig 5 below shows a data flow of the proposed algorithm. Like other metaheuristic algorithms, the proposed algorithm starts with initial birthing lairs containing seal pups. To make the terminology clear and easy, we can use the following simple terms. Each lair represents a solution. The quality of the lairs represents the quality of the solution, and thus the suitability of the lair for seal pupping.

The RSS in this paper can be described as an iterative algorithm based on population. Despite other population-based algorithms such as GA, where the reproduction of new generations ensures generating new solutions, the RSS is based only on seal pups life cycle. As all population based algorithms, RSS starts with an initialized number of lairs.

Certain studies about asymptotic probability convergence theories considering the underlying operations which are characterized by a Markov nature, requires to be balanced, and thus resulting in the algorithm wasting a lot of its efficiency. The power of stochastic algorithms mainly is based on the fact that the probabilistic nature of the algorithms guarantee that the algorithms do not necessarily get trapped at local optima.

The RSS consists of two search states that alternate randomly via the noise emitted by predators. This can provide a balance between exploitation and exploration of the search, and thus the probability to get local optima easily is very low.

In the following section, RSS is tested and validated using 15 benchmark optimization problems (benchmark functions). Then, a comparison with GA, PSO and CS is presented.

## Experimental Results

A comprehensive set of 15 test functions, collected from references [10,17,19,20,71–75] were used to test the performance of the proposed algorithm. Table 1 presents the benchmark test functions used in this experimental study. According to the references mentioned above, the selected functions fulfil the requirements of uni-objective and multi-objective problems. It is very important to highlight that the main target of this benchmarking test is to check whether the proposed algorithm RSS is able to solve uni-objective and multi-objective optimization problems. The values of *n* represent the dimension of the problem (function), f_*_ indicates the optimum value of the test function, and *S* indicates the search space bounds. The optimum values of the functions *F*_1_,*F*_4_,*F*_6_,*F*_9_,*F*_10_,*F*_11_,*F*_12_,*F*_13_,*F*_15_ is at f_*_ = 0, for *F*_2_ is at f_*_ = −1, for *F*_3_ is at f_*_ = −418.982*n*, for *F*_5_ is at f_*_ = −1.8013, for *F*_7_ is at f_*_ = −4.6877, for *F*_8_ is at f_*_ = −186.730, for *F*_14_ is at f_*_ = −186.730.

**Table 1:** List of benchmark test functions.

Test function	*F*_*id*	*S*	f_*_	*n*
$f\left(x\right)=\sum_{i=1}^{n}x_{i}^{2}$	*F*_1_	[−5.12,5.12]	0	30
*f*(*x*,*y*) = −cos(*x*)cos(*y*)exp[−(*x*−*π*)^2^−(*y*−*π*)^2^]	*F*_2_	[−100,100]	−1	30
$f\left(x\right)=\sum_{i=1}^{n}\left[-x_{i}\sin\left(\sqrt{\left|x_{i}\right|}\right)\right]$	*F*_3_	[−500,500]	−418.98*n*	30
$f\left(x\right)=-20.\exp\left(\sqrt{\frac{1}{n}}\sum_{i=1}^{n}x_{i}^{2}\right)-\exp\left(\sqrt{\frac{1}{n}}\sum_{i=1}^{n}\cos\left(2\pi x_{i}\right)+\left(20+e\right)\right)$	*F*_4_	[−32.76,32.76]	0	30
$f\left(x,y\right)=-\sin\left(x\right)\sin^{2m}\left(\frac{x^{2}}{\pi }\right)-\sin\left(y\right)\sin^{2m}\left(\frac{2y^{2}}{\pi }\right)$	*F*_5_	[0,5]	−1.8013	30
$f\left(x\right)=10n+\sum_{i=1}^{n}\left[x_{i}^{2}-10\cos\left(2\pi x_{i}\right)\right]$	*F*_6_	[−5.12,5.12]	0	30
$f\left(x\right)={\sum_{i=1}^{n}\sin\left(x_{i}\right)\left[\sin\left(\frac{ix_{i}^{2}}{\pi }\right)\right]}^{2m}$	*F*_7_	[0,*π*]	−4.6877	30
$f\left(x,y\right)=\sum_{i=1}^{5}i\cos\left[\left(i+1\right)x+1\right]\sum_{i=1}^{5}\cos\left[\left(i+1\right)y+1\right]$	*F*_8_	[−10,10]	−186.730	30
$f\left(x,y\right)=\sum_{i=1}^{n-1}\left[{\left(1-x_{i}\right)}^{2}+100{\left(x_{i+1}-x_{i}^{2}\right)}^{2}\right]$	*F*_9_	[−10,10]	0	30
$f(x)=\frac{1}{4000}\sum_{i=1}^{n}x_{i}^{2}-\prod_{i=1}^{n}\cos\left(\frac{x_{i}}{\sqrt{i}}\right)+1$	*F*_10_	[−600,600]	0	30
$f\left(x\right)={\left(x_{1}-1\right)}^{2}+\sum_{i=2}^{d}i{\left(2x_{i}^{2}-x_{i-1}\right)}^{2}$	*F*_11_	[−10,10]	0	30
$\begin{array}{l} f\left(x\right)=\sin^{2}\left(\pi \omega_{1}\right)+{\sum_{i=1}^{d-1}\left(\omega_{i}-1\right)}^{2}\left[1+10\sin^{2}\left(\pi \omega_{i}+1\right)\right]+\\ {\left(w_{d}-1\right)}^{2}\left[1+\sin^{2}\left(2\pi \omega d\right)\right] \end{array}$	*F*_12_	[−10,10]	0	30
$f(x)={\sum_{i=1}^{d}\left(\sum_{j=1}^{d}\left(j^{i}+\beta \right)\left({\left(\frac{x_{i}}{j}\right)}^{i}-1\right)\right)}^{2}$	*F*_13_	[−*d*,*d*]	0	30
$f(x)=\left(\sum_{i=1}^{5}i\cos\left(\left(i+1\right)x_{1}+i\right)\right)\left(\sum_{i=1}^{5}i\cos\left(\left(i+1\right)x_{2}+i\right)\right)$	*F*_14_	[−10,10]	−186.730	30
$f\left(x\right)=\sum_{i=1}^{n}x_{i}^{2}$	*F*_15_	[−5.12,5.12]	0	30

**Fig 6:**
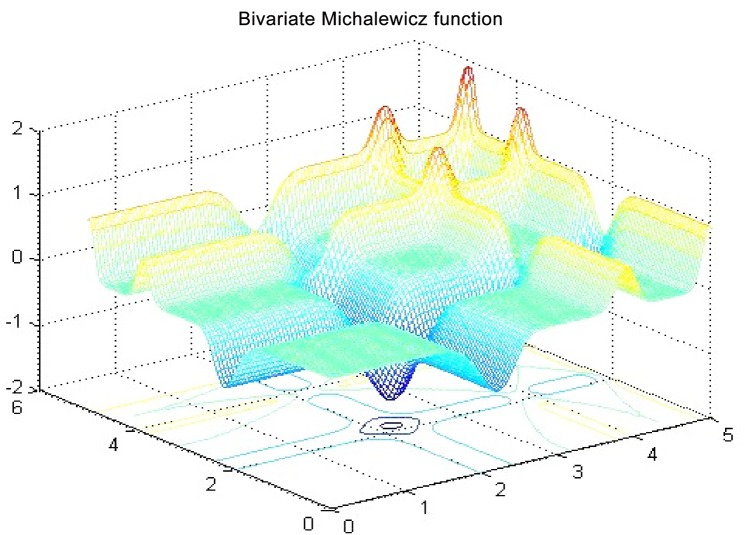
*F*_5_ function in 2D for *m* = 10, *n* = 5.

A description of the test function *F*_5_ (Bivariate Michalewicz) is presented in Fig 6 (for other test functions: refer to Figs A-N in S1 Appendix). It is considered as a multi-objective function, which have *n* local optima. This function is characterized by the parameter *m* that defines the ruggedness (steepness) of the valleys. The setting of *m* with a large value conducts to uneasy search. When the *m* value is very large, the functions perform as a needle in the haystack, something that is so difficult to find, especially because the area of search is too large.

**Fig 7:**
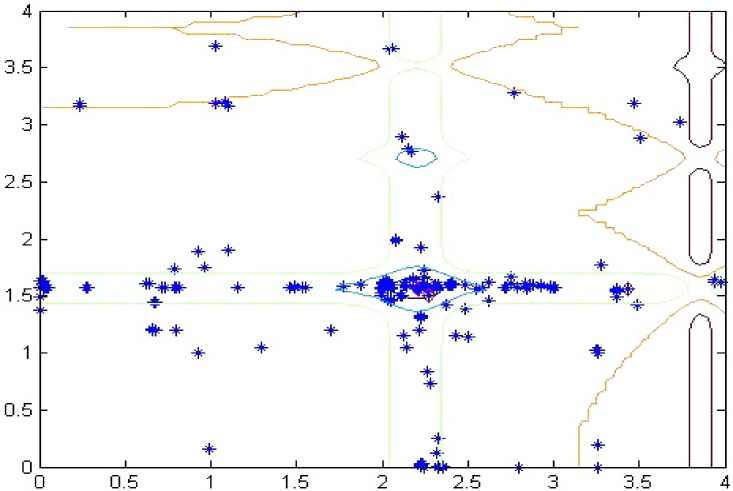
Searching for a new solutions by using Ringed Seal Search, final achieved solutions are highlighted with a diamond.

The search area is bounded to a hypercube, where (*x*,*y*) ∈ [0,5]×[0,5],*i* = 1,⋯,*n*, and the value of m = 10. The global minimum is approximated by f_*_ = −1.8013 for *n* = 2. The landscape of the function *F*_5_ is equation is described in Fig 7.

From Fig 7, we can notice that the used lairs are converging towards the global optimum. The figure also shows that the lairs are distributed at different local optima. This feature demonstrates the ability of RSS to deal with multi-objective problems and escape local optima traps. Escaping from local optima is particularly related to the optimal balance between exploitation and exploration, which is realized via the sensitive search model. As a result, we can conclude that modeling of the external noise via a uniform distributed pseudorandom integer is efficient for the imitation of the switch between normal state and urgent state.

In order to confirm the efficiency of the sensitive search model to deal with uni-objective and multi-objective problems, a series of simulations were tested with a varied number of lairs: *l* = 5 until *l* = 200. The results show that an efficient result for the majority of the optimization problems is achieved when *l* = 10. The results also show that the convergence is not affected by changing the parameters values. The following section introduces the performance of the proposed algorithm to other meta-heuristic algorithms based on the standard problems (test functions).

### Performance Comparison with Other Meta-Heuristic Approaches

We have applied the RSS algorithm to fifteen test function whose results have been compared to those obtained by the GA, the PSO and the CS. These algorithms are considered as the most popular baseline approaches in many optimization applications [10,11,18,20,23,76]. In order to make the comparison more valuable CS was selected. CS is considered as a hill climbing variant that comprises the brood parasitic behaviour of cuckoo birds, and it uses Levy flights to enhance the balance between exploitation and exploration of the search space [10]. The overall comparison, and the parameters of each algorithm were set to be compatible with their original setting. The maximum number of iterations for the test functions in Table 2 and Table 3 was set to 100. These criterion also have been selected to fulfil the requirements of similar works highlighted in the literature.

**Table 2:** AB, MB and SD results using uni-Objective functions.

Function ID		GA	PSO	CS	RSS
***F***_***1***_	AB	0.2096	0.2096	0.1584	0.1516
	MB	0.0537	0.0537	0.9852	0.9472
	SD	0.0360	0.0360	0.1858	0.1850
***F***_***2***_	AB	-0.9934	-0.9934	-0.6262	-0.6299
	MB	-1.0000	-1.0000	-0.8049	-0.8141
	SD	0.0660	0.0660	0.3894	0.3892

**Table 3:** Var and SQ results using uni-Objective functions.

Function ID		GA	PSO	CS	RSS
***F***_***1***_	Var	0.0012	0.0012	0.0345	0.0342
	SQ	0.0257	0.0001	0	0
***F***_***2***_	Var	0.0043	0.0043	0.1516	0.1514
	SQ	-0.0377	-0.9724	-0.7039	-0.9962

#### Parameters Setting

The parameters setting for each algorithm during this comparison is described as below:

1. Genetic Algorithm: the parameters of GA are set to *G*_*i*_ = 100 and the population size *α* = 20; where the total number of iterations is set to 100 for all the test functions.
2. Particle Swarm Optimization: the velocity, social and cognitive parameters are set to 2.
3. Cuckoo Search: The parameters consist of the number of the nest, which is set to 15 nests, and the rate of detection *p*_*α*_ = 0.25.
4. Ringed Seal Search: Two parameters have been tuned up in RSS: the mortality rate of the seal pups: *rate* = 15%, and the initial number of the birthing lairs: *l* = 10.

The experimental comparisons between these meta-heuristic algorithms with the proposed RSS were developed according to the type of the test function: uni-objective such as *F*_1_ and *F*_2_, or multi-objective such as: *F*_3_, *F*_4_, *F*_5_, *F*_6_, *F*_7_, *F*_8_, *F*_9_, *F*_10_, *F*_11_, *F*_12_, *F*_13_, *F*_14_ and *F*_15_. The reason behind choosing only two uni-objective functions is that uni-objective problems are easy to solve (the landscape is not complex). In some literatures it is called smooth problem containing only one global optimum. In contrast, the multi-objective problems were tested with 8 test functions representing variant complex problems. The test consists of comparing the RSS to other algorithms such as GA, PSO and CS. The reported results in the next sections are featured with the following performance indexes during 100 iterations: the Average Best-so-far (AB) solution, the Median Best so-far (MB), the Standard Deviation (SD) of the best-so-far solution, the Variance (Var) of the best-so-far solution and the Solution Quality (SQ) for each function. During each run the best value of *M* is saved; thus during 100 times run, 100 best values are produced. The Average best is computed from the mean of 100 best values. The Median best is the midpoint of 100 best values. The SD is the standard deviation of 100 best values and the Var is just the square of the SD. The mathematical formulations are defined as below:

$$AB=\frac{{\sum_{i=1}^{n}f_{best}}_{i}}{n}$$ (8)

$$SD=\sqrt{\frac{\sum_{i=1}^{n}{\left(f_{best\;i}-AB\right)}^{2}}{n-1}}$$ (9)

$$MB=f_{best\;k},\;\text{where}\;k=\left(\frac{n+1}{2}\right)$$ (10)

where *f*_*best i*_ is the best value of each run and *n* is the number of run.

#### Uni-objective test functions

This experiment was applied on uni-objective test functions *F*_1_ and *F*_2_. The obtained results are presented in the following table.

From Table 2, the proposed algorithm RSS performs better than CS, PSO and GA for both *F*_1_ and *F*_2_. For RSS, this can be interpreted as a fast convergence to the optimum better than other algorithms. Moreover, this difference in performance is related to the trade-off between exploration and exploitation during the search for the optimum. This trade-off can be seen in the reported values of AB, MB and SD. For more performance tests, we calculate the variance to measure the dispersion of the achieved solutions and the solution quality to measure the capability to find global optimum values.

From Table 3, the variance and the solution quality results confirm the trade-off between exploitation and explorations during the search for solutions. The variance values of RSS indicate that there is a dispersion in terms of the achieved solutions with a value Var = 0.0342 for *F*_1_ and a value Var = 0.1514 for *F*_2_. Furthermore, the quality solution values for RSS were equal to the global optimum values of the corresponding functions which demonstrate that RSS is able to search and find global optimum values.

The experimental results obtained from uni-objective test functions show that RSS achieved the global optimum easily. On the other hand the comparison shows that there is no significant difference with other approaches. This is related to the nature of the problems which are considered as smooth problems which are easy to solve compared to multi-objective problems which are more complex.

#### Multi-objective test functions

These functions represent a complex benchmark test as they contain various local optima. In such multi-objective problems, the results reflect the ability of the proposed algorithm to escape local optima traps. The results are averaged over 100 iterations, reporting the performance index for each function as shown in Table 4.

**Table 4:** AB, MB and SD results using multi-objective functions.

Function ID		GA	PSO	CS	RSS
***F***_***3***_	AB	-820.7881	-718.5607	-827.2707	-831.1258
	MB	-831.4344	-719.5274	-837.6642	-837.9523
	SD	24.5092	117.2421	29.5045	26.0190
***F***_***4***_	AB	3.8122	0.4933	0.0270	0.0105
	MB	3.7101	0.0279	0.0123	0.0028
	SD	2.0933	1.5303	0.0360	0.0259
***F***_***5***_	AB	-1.5819	-1.5933	-1.6026	-1.6026
	MB	-1.5906	-1.6026	-1.6026	-1.6026
	SD	0.0228	0.0526	0	0
***F***_***6***_	AB	1.3062	9.8941	0.1493	0.0159
	MB	1.1959	3.6502	0.0135	0.0052
	SD	0.9546	11.1731	0.3092	0.0216
***F***_***7***_	AB	-1.7725	-1.7847	-1.8013	-1.8013
	MB	-1.7878	-1.8013	-1.8013	-1.8013
	SD	0.0443	0.0928	0	0
***F***_***8***_	AB	-181.0069	-79.1157	-209.2064	-210.1891
	MB	-185.0235	-27.8028	-210.3642	-210.4089
	SD	26.0941	109.9932	3.7531	0.8273
***F***_***9***_	AB	0.1736	16.9143	0.0208	0.0033
	MB	0.0964	0.3611	0.0009	0.0001
	SD	0.1782	72.6749	0.0452	0.0093
***F***_***10***_	AB	0.6182	1.3113	0.0223	0.0173
	MB	0.6226	1.0656	0.0215	0.0149
	SD	0.3043	1.4230	0.0140	0.0123
***F***_***11***_	AB	0.3577	6.2987	0.0001	0
	MB	0.1872	0.0001	0	0
	SD	0.5382	31.7590	0.0004	0
***F***_***12***_	AB	0.0467	0.0035	0	0
	MB	0.0248	0	0	0
	SD	0.0527	0.0214	0	0
***F***_***13***_	AB	0.7116	10.1861	0.0002	0
	MB	0.3604	0.0341	0	0
	SD	0.8461	39.4115	0.0007	0.0001
***F***_***14***_	AB	-7.0474	-8.5533	-11.0052	-11.0289
	MB	-5.2529	-11.0297	-11.0288	-11.0309
	SD	2.9045	4.3184	0.0616	0.0052
***F***_***15***_	AB	-177.8809	-95.2011	-186.6910	-186.6111
	MB	-180.4464	-137.1709	-186.7237	-186.6907
	SD	9.3851	92.5425	0.0987	0.1759

From Table 4, AB, MB and SD results show that RSS outperforms other algorithms in most of the functions. The superiority of RSS over CS, PSO and GA can be seen in *F*_3_, *F*_4_, *F*_6_, *F*_8_, *F*_10_, *F*_11_, *F*_13_ and *F*_14_ where RSS was able to reach the global optimum during 100 iterations better than other algorithms. For example, in *F*_6_ RSS could reach an AB of 0.0159, by contrast CS only achieved 0.1493, PSO achieved 9.8941 and GA achieved 1.3062. In the following table the variance and the solution quality were measured for this category of functions in order to get more evidences about the outperformance of RSS.

From Table 5, the variance and solution quality values demonstrate the superiority of RSS compared to other algorithms. This can be justified by the diversification and the optimal balance between exploration and exploitation by RSS during the search for the global optimum. As a consequence, the RSS shows an outstanding capability to search and find new positions with quality solution equal to the expected global optimums.

**Table 5:** Var and SQ results using multi-objective functions.

Function ID		GA	PSO	CS	RSS
***F***_***3***_	Var	600.7000	13745.71	870.5155	676.9883
	SQ	-812.2354	-765.3373	-831.8597	-832.6857
***F***_***4***_	Var	4.3819	2.3418	0.0012	0.0006
	SQ	3.9664	0.7778	0.0171	0.0072
***F***_***5***_	Var	0.0005	0.0028	0	0
	SQ	-1.5317	-1.5209	-1.6026	-1.6026
***F***_***6***_	Var	0.9112	124.8382	0.0956	0.0005
	SQ	1.1525	4.3005	0.1450	0.0174
***F***_***7***_	Var	0.0020	0.0086	0	0
	SQ	-1.7737	-1.7181	-1.8013	-1.8013
***F***_***8***_	Var	0.0681	1.2099	0.0014	0.0001
	SQ	-167.0768	-88.2854	-210.2348	-209.8650
***F***_***9***_	Var	0	5.2816	0	0
	SQ	0.1694	106.0209	0.0218	0.0052
***F***_***10***_	Var	0.0926	2.0248	0.0002	0.0002
	SQ	0.6132	0.7230	0.0185	0.0169
***F***_***11***_	Var	0.0003	1.0086	0	0
	SQ	0.3733	0.2911	0.0002	0
***F***_***12***_	Var	0.0028	0.0005	0	0
	SQ	0.0591	0	0	0
***F***_***13***_	Var	0.0007	1.5533	0	0
	SQ	0.2663	5.0913	0.0001	0
***F***_***14***_	Var	8.4359	18.6484	0.0038	0
	SQ	-7.5363	-8.2977	-10.3955	-11.0303
***F***_***15***_	Var	0.0881	8.5641	0	0
	SQ	-176.5184	-117.7560	-186.6132	-186.6440

The balance between exploitation and exploration during the search is achieved through the sensitive search model, where the seal pup can take two different states: urgent or normal. It is very important to highlight the importance of the obtained results in term of measuring the ability of RSS to escape poor local optima traps and its ability to locate a near-global optimum. For the multi-objective functions *F*_3_ to *F*_15_ are quite pertinent as the number of their local minima increases once their dimensions *n* increase.

From Tables 2, 3, 4 and 5, we can conclude that RSS, CS, PSO and GA were able to find the global optimum during 100 iterations. However, RSS shows an outperformance in terms of the average best solutions and also the solution quality in most of the test functions. Furthermore, the measurement of the standard deviation, median best and variance clarified how RSS search is diversified. This diversification is the fruit of the optimal balance between exploration and exploitation. Another advantage for RSS can be seen in the high quality solutions which are equal or approximately near the global optimum. In the following figures we explain how RSS algorithm consumed less number of iterations to achieve the global optimum.

**Fig 8:**
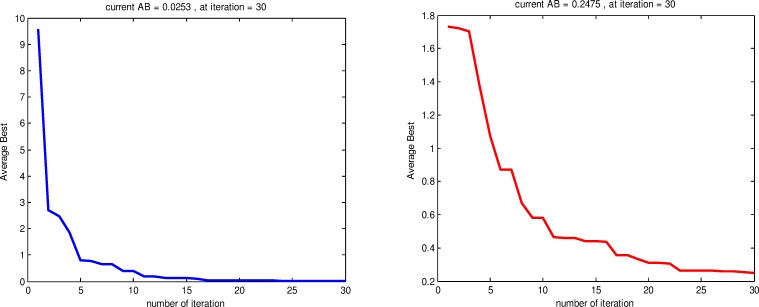
Average Best convergence of *F*_9_ test function using RSS (a) and CS (b).

**Fig 9:**
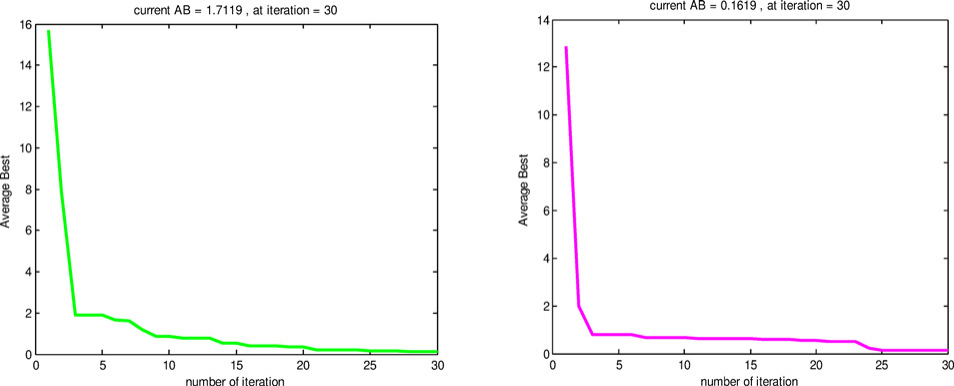
Average Best convergence of *F*_9_ test function using PSO (a) and GA (b).

Figs 8 and 9 show a sample convergence rate plot of *F*_9_ function using RSS, CS, PSO and GA during 30 iterations. As we can see in Fig 8 (A), RSS has reached a global minimum at the 17^th^ iteration and the obtained AB value is 0.7119. On the other hand, with a same landscape problem, in Fig 8 (B), CS algorithm approximately reached a global minimum at the 30^th^ iteration with a value AB equal to 0.1619.

The Convergence rate of *F*_9_ function using PSO achieved a global minimum at 25^th^ iteration and the AB value is 0.3016 as shown in Fig 9 (A). On the other hand, Fig 9 (B) shows GA reached a global minimum at 30^th^ iteration and the AB value is 0.1619. In conclusion, the *F*_9_ test function shows how the RSS converged quickly to the global optimum compared to PSO, GA and CS. For more test results, we applied GA, PSO, CS and RSS on all the test functions mentioned in Table 1 to compare the average best for each algorithm.

**Fig 10:**
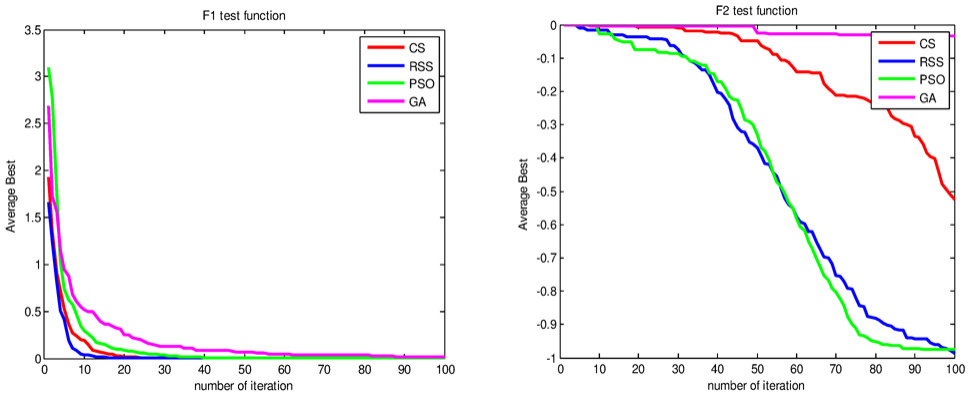
AB convergence of CS, RSS, PSO and GA for minimization of (a) *F*_1_ and (b) *F*_2_.

**Fig 11:**
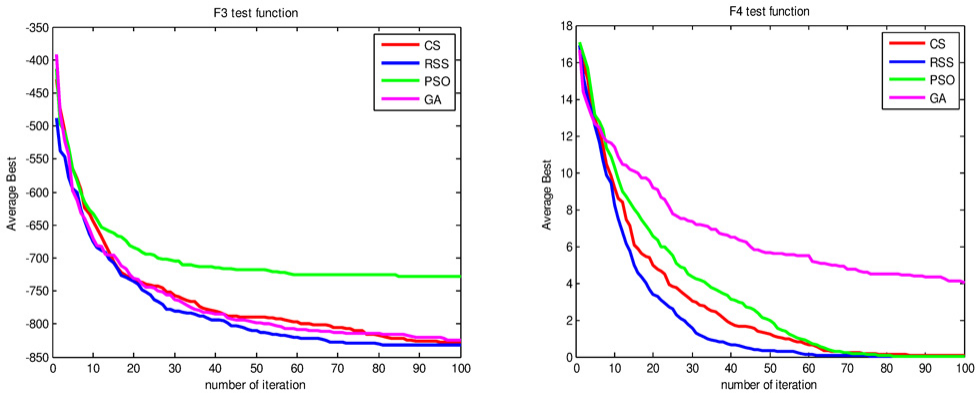
AB convergence of CS, RSS, PSO and GA for minimization of (a) *F*_3_ and (b) *F*_4_.

**Fig 12:**
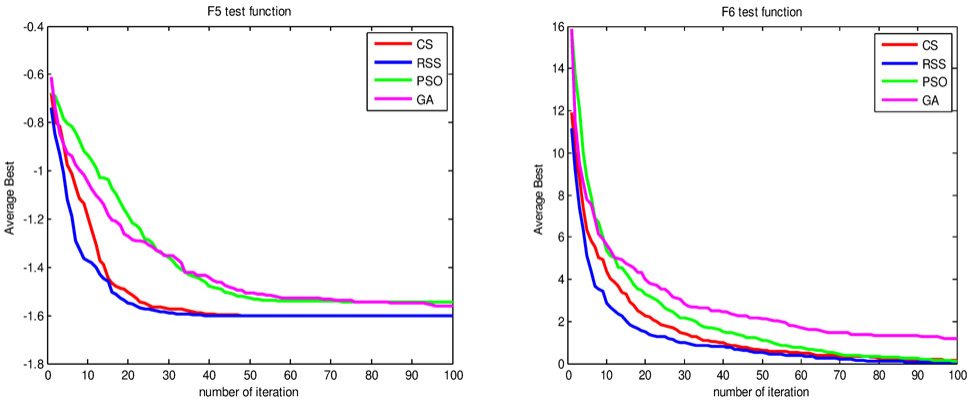
AB convergence of CS, RSS, PSO and GA for minimization of (a) *F*_5_ and (b) *F*_6_.

**Fig 13:**
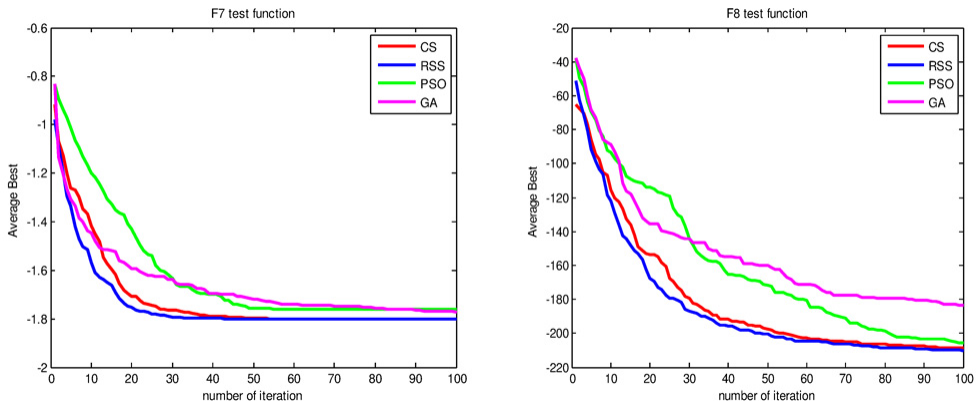
AB convergence of CS, RSS, PSO and GA for minimization of (a) *F*_7_ and (b) *F*_8_.

**Fig 14:**
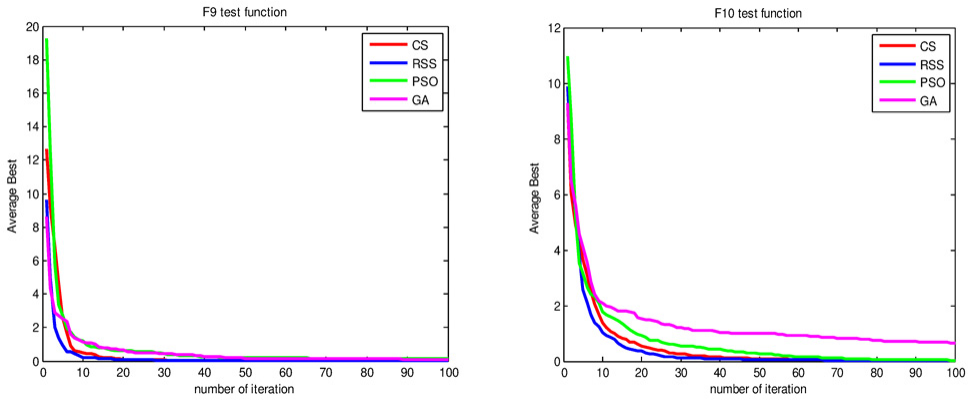
AB convergence of CS, RSS, PSO and GA for minimization of (a) *F*_9_ and (b) *F*_10_.

**Fig 15:**
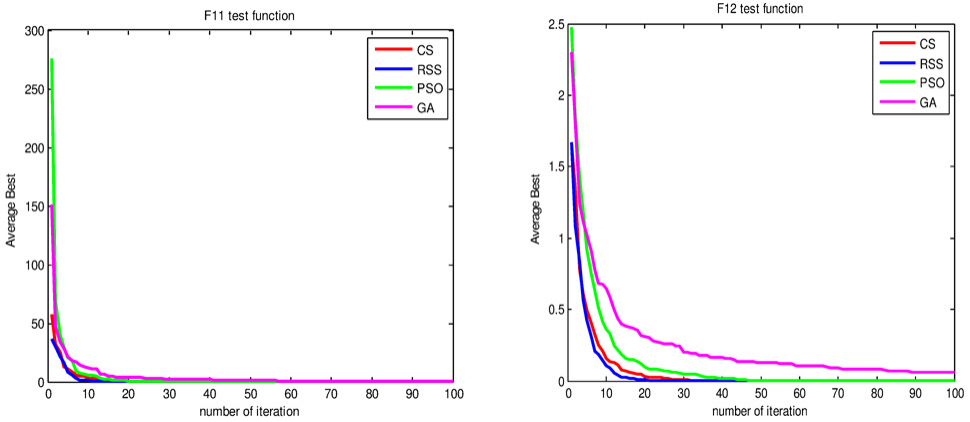
AB convergence of CS, RSS, PSO and GA for minimization of (a) *F*_11_ and (b) *F*_12_.

**Fig 16:**
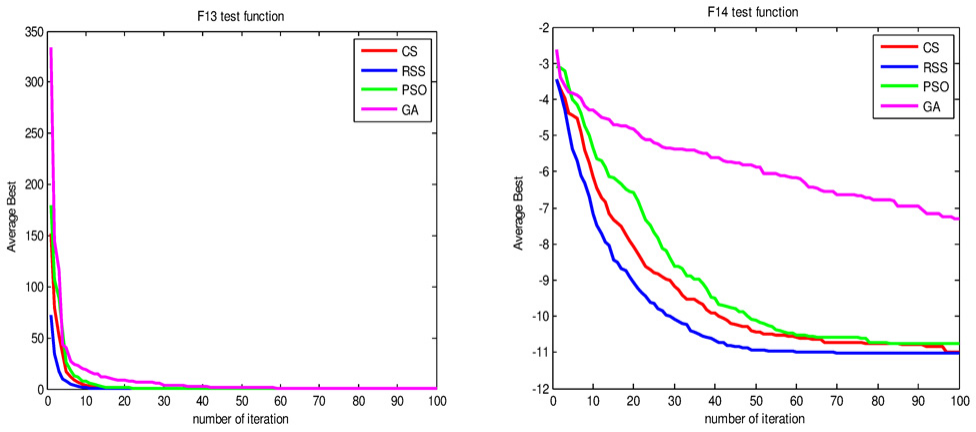
AB convergence of CS, RSS, PSO and GA for minimization of (a) *F*_13_ and (b) *F*_14_.

**Fig 17:**
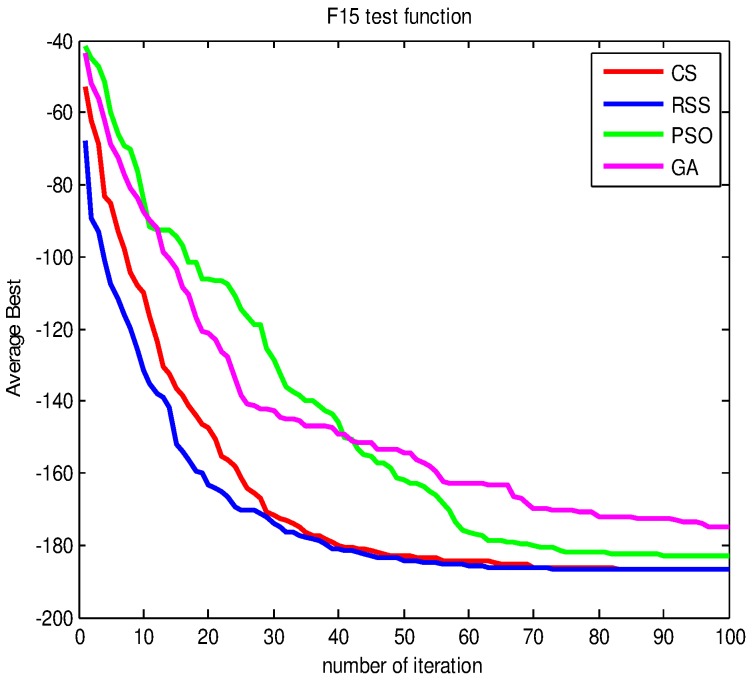
AB convergence of CS, RSS, PSO and GA for minimization of *F*_15_.

Figs 10–17 present the convergence rates based on the average best outputs for GA, PSO, CS and RSS algorithms considering the functions *F*_1_, *F*_2_, *F*_3_, *F*_4_, *F*_5_, *F*_6_, *F*_7_, *F*_8_, *F*_9_, *F*_10_, *F*_11_, *F*_12_, *F*_13_, *F*_14_ and *F*_15_. Thus, the smaller the number of required steps, the higher the convergence speed. The results show that RSS consumes less time to reach the global optimum. This is a proof that RSS outperforms GS, PSO and CS in terms of convergence to the global optima. The evidence shows how RSS quickly converged to the global optimum compared to other algorithms. During little iteration RSS can have the ability to reach the global optimum. This can be justified by the optimal exploitation-exploration based on the sensitive search model inspired from seal movement. It is worth noting that, faster convergence does not necessarily mean an optimal output. In fact, too fast convergence may lead to the problem of prematureness, which leads the search to be trapped at local optima positions. As shown in Table 3 and Table 5, the SQ values demonstrate that the final achieved positions are equal or quite near the optimal values. This is a proof that the RSS mechanism escaped local optima traps.

To uncover the underlying mechanism of our algorithm, we examined the optimization process in terms of variance point of view. For the sake of simplicity, in the following we will present the results for the function *F*_15_. The results for other functions are alike and not shown here.

**Fig 18:**
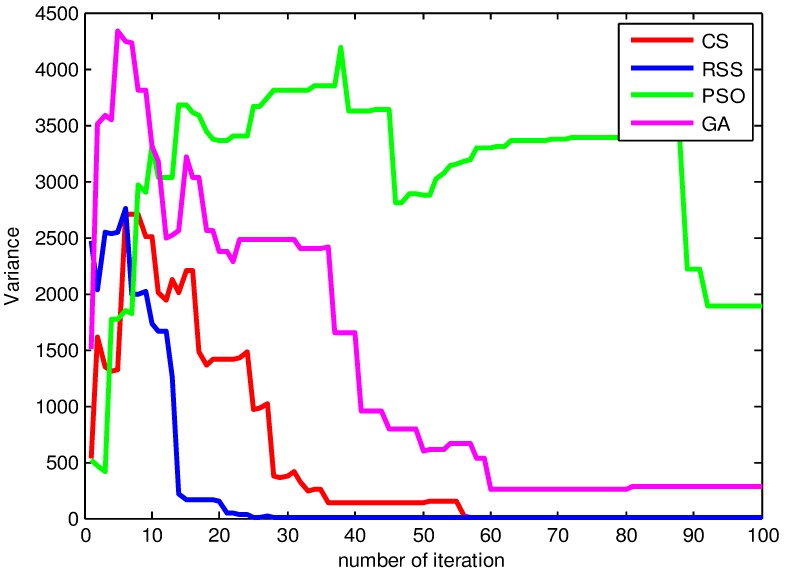
The variance values during different iterations of CS, RSS, PSO and GA for *F*_15_.

From Fig 18, the variance values of RSS confirm the AB convergence results found previously. This finding is related to the fact that the search dispersion is in harmony with the convergence which can be seen in the smaller values of variance at certain level of iterations. For example, starting from iteration = 24 the RSS achieves a variance Var = 0. This can be justified by the reason that RSS search has already reached or quite near the global optimum. Taking into account the variance values during the first 20 iterations, we can conclude that the variance has been influenced by the search findings and the focus on the positions with high fitness values reduces the dispersion of the search positions gradually until the best optimal position is found.

In the following section, a case study based on FPGA platform is implemented in order to show the significance of using RSS algorithm.

### FPGA Implementation

**Fig 19:**
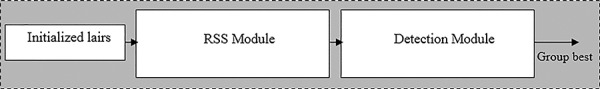
Configuration of the RSS system.

In this section, we present the implementation of RSS on FPGA. Therefore, the results are compared with those obtained by Simulated Annealing (SA) [77,78]. FPGAs are semiconductor devices based on a matrix of configurable logic blocks (CLBs), which are connected to each other via programmable interconnects. The main feature of FPGAs is the capability to be reprogrammed to specific application or functionality requirements after manufacturing. FPGAs are used for a vast range of applications in science and technology, such as therapy applications in medicine, aerospace applications, wired communications applications, multimedia and safety systems. It has also been shown that FPGAs are suitable for the implementation of soft computing applications [79–81]. At high level, the mapping of the RSS algorithm into the corresponding FPGA design is straightforward as shown in Fig 19. It consists of two main modules: RSS module and detection module.

### RSS Module

**Fig 20:**
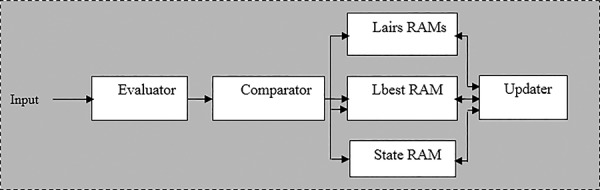
The block diagram of the RSS module.

A block diagram of the hardware implementation of the RSS algorithm can be seen in Fig 20. The design is divided into four main blocks: an evaluator block, comparator block, RAM block and an update block.

In this system, we used 10 lairs (initial candidate solutions). These lairs work in parallel to achieve the targets of the objective function.

#### The Evaluator

The evaluator calculates the fitness value. In this paper, we chose the Rastrigin function as a fitness function. It is described as below:

$$f\left(x\right)=16+\sum_{i=1}^{2}\left(x_{i}^{2}-8\left(1-4x_{i}^{2}+\frac{1}{4}x_{i}^{4}-\frac{1}{128}x_{i}^{6}+\frac{1}{8192}x_{i}^{8}\right)\right)$$ (11)

This test function is assumed with an optimal value of 0 and 8th order. The RSS searcher is required to find the optimal point (0.0 0.0) in the range [-5.5, 5.5]. A zero is set as the optimal value of this multi-objective objective problem. Fig C in Appendix A shows the shape of this function.

#### The Comparator

The comparator block consists of comparing the current fitness value with the best fitness value. In case where the current fitness value is better than the global fitness value, the global fitness value is updated to be equal to the current. Then, the comparator block provides a signal to the Lbest RAM and stores the current position in Lairs RAM.

#### The Memory Block

The memory block constitutes three RAMS: Lairs RAM, Lbest RAM and State RAM. The Lairs RAM is used to store the position of the lairs, the Lbest RAM stores the best lairs values and the State RAM stores the current state of the RSS searcher.

#### The Updater Block

The lairs position and states are updated periodically until the best solution is achieved. The initial values of the update block come from the RAM block. After computation, the position and the state values are then delivered back to the RAM.

### The Detection Module

The role of the detection module consists of finding and storing the best estimated value for each lair. The module searches the best lair ever found, called group best in this paper. Then, the lairs’ best values and the group best value are stored in the RAM module.

### Simulation Results

The simulation results in this paper are obtained using the Rastrigin test function. In this simulation, 10 lairs were used as candidate solutions. These lairs work in parallel to achieve the optimal value of the objective function. The obtained results are compared with those obtained by SA. Both RSS and SA were tested based on the objective function to see which one performs better in terms of achieving the optimal value 0.

The parameters setting of RSS and SA algorithm during this simulation are described as below:

1. Simulated Annealing: Hard Limit *HL* = 20, Soft Limit *SL* = 30, maximum temperature *T*_max_ = 100, minimum temperature *T*_min_ = 0.01.
2. Ringed Seal Search: Only one parameter has been tuned up in RSS: The initial number of the birthing lairs: *l* = 10. However, the mortality rate of the seal pups is set by default: *rate* = 15%.

**Fig 21:**
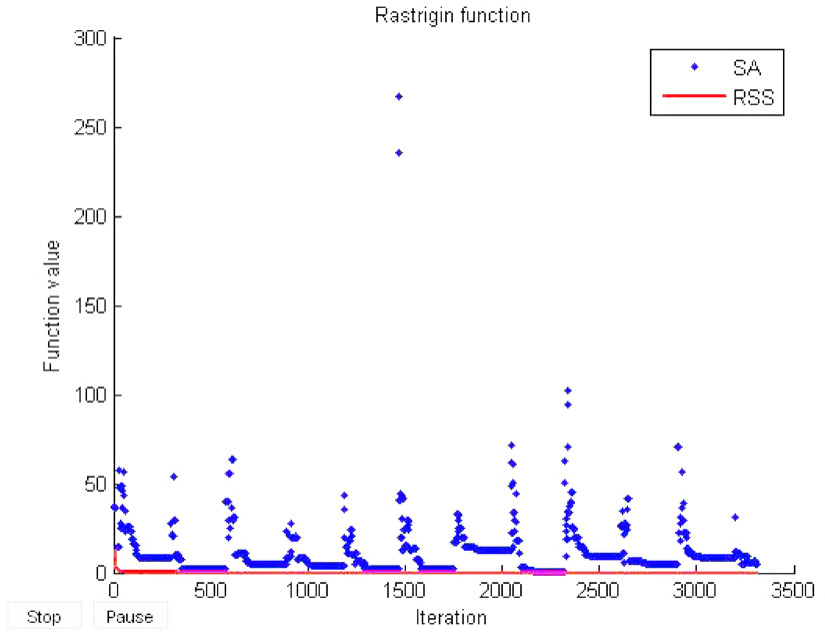
Evaluation function of RSS compared with SA.

Fig 21 shows the evaluation function of RSS compared to SA. Both of the algorithms were able to reach the optimum value 0. However, RSS outperformed SA in terms of fast convergence to the global optimum. The convergence of RSS started almost from the 50^th^ iteration. In contrast, SA consumed more time, almost 300 iterations to start the convergence. The outperformance of RSS can be interpreted by the optimal balance used by RSS during search.

This result also shows that the FPGA system based on RSS can easily find the optimum with 10 lairs as candidate solutions. After almost 50 iterations, the found lairs were very close to the global optimum. As a consequence, this comparison demonstrates the significance of using RSS to solve optimization problems for FPGA platforms.

## Contribution

The contributions of this work are underlined as below:

1. The main component in the proposed algorithm RSS is the Sensitive Search Model which is inspired by the ringed seal behavior. This model is used by RSS to get an optimal balance between exploitation and exploration of the search.
2. The *search* in RSS is inspired by the ringed seal search, which is able to solve uni-objective and multi-objective problems with high performance compared to other popular algorithms such as GA, PSO and CS.
3. The number of parameters to tune for the proposed algorithm RSS is only one parameter, making RSS to be less sensitive to parameters settings compared to CS with two tuning parameters.

## Significance

The proposed RSS algorithm is able to solve uni and multi-objective optimization problems. The significance of such algorithms can be summarized into two main points:

1. From a heuristic search point of view, RSS algorithm represents an innovative way on how *searchers* move in the search space to find global optimum values. Moreover, this paper constitutes the second attempt in metaheuristic algorithms that addresses the problem of optimal balance between exploration and exploitation after a first attempt presented by Yang et al [16].
2. From optimization applications point of view, the results obtained in this paper show that RSS has the potentials to be used in solving problems such as cancer classification applications[82], optimization of web service composition processes [83], vehicle routing system applications [84], design of embedded systems [85], collective robotic search applications [86], data clustering applications [87–90], digital games applications [91], medical images applications [92], etc.

## Conclusions

In last recent years, several metaheuristic optimization algorithms have been introduced. The main idea consists of imitating a natural phenomenon that has existed on the earth since millions of years. Typically, these phenomena strategy consists of two parameters: a population parameter, and the movement inside the search area. In this paper, we presented a novel nature-inspired algorithm for global optimization called Ringed Seal Search (RSS). It is inspired by the amazing search behavior of seal’s pups. The search is characterized by an optimal balance between exploitation-exploration of the search based on the sensitive behavior of seals. A sensitive search is modeled, where the pup can be in normal state when there is no external noise, or urgent state in case of external noise. In normal state the seal pup performs a Brownian walk inside the local area. However, during urgent state the seal pup leaves the proximity and performs Levy walk to find other solutions. This taxonomy expected to fulfill the requirements of intensification (exploitation) and diversification (exploration).

RSS was experimentally tested considering a suite of fifteen benchmark test functions. The performance of RSS was also evaluated in terms of convergence rate to the global optimum compared to GA, PSO and CS. The results confirmed that RSS outperforms other algorithms in terms of fast convergence rate to the global optimum.

The RSS outperformance of GA, PSO and CS is associated with two reasons:

1. The division of the search pattern into two states (normal and urgent) provided a strong mechanism to model an optimal balance between exploitation and exploration.
2. The number of parameters to tune for RSS is less as compared with other algorithms such as PSO and GA.

The RSS is able to be used to study multi-objective problems including NP-hard problems. Seal sensitivity, point to point random trajectory for the seal are features that deserve further focus in future research. Moreover, further studies will be conducted in the future to study Pareto fronts for trade-off solutions generated by the RSS. We also believe that this work can accept different modification based on the behavior of seals.

## Supporting Information

S1 Appendix The landscaped of the test benchmark functions. (ZIP)
