# Supplementary figures and images for: Ringed Seal Search for Global Optimization via a Sensitive Search Model
Source: PLoS One. 2016 Jan 20;11(1):e0144371. doi: 10.1371/journal.pone.0144371 (PMC4720396; doi:10.1371/journal.pone.0144371)

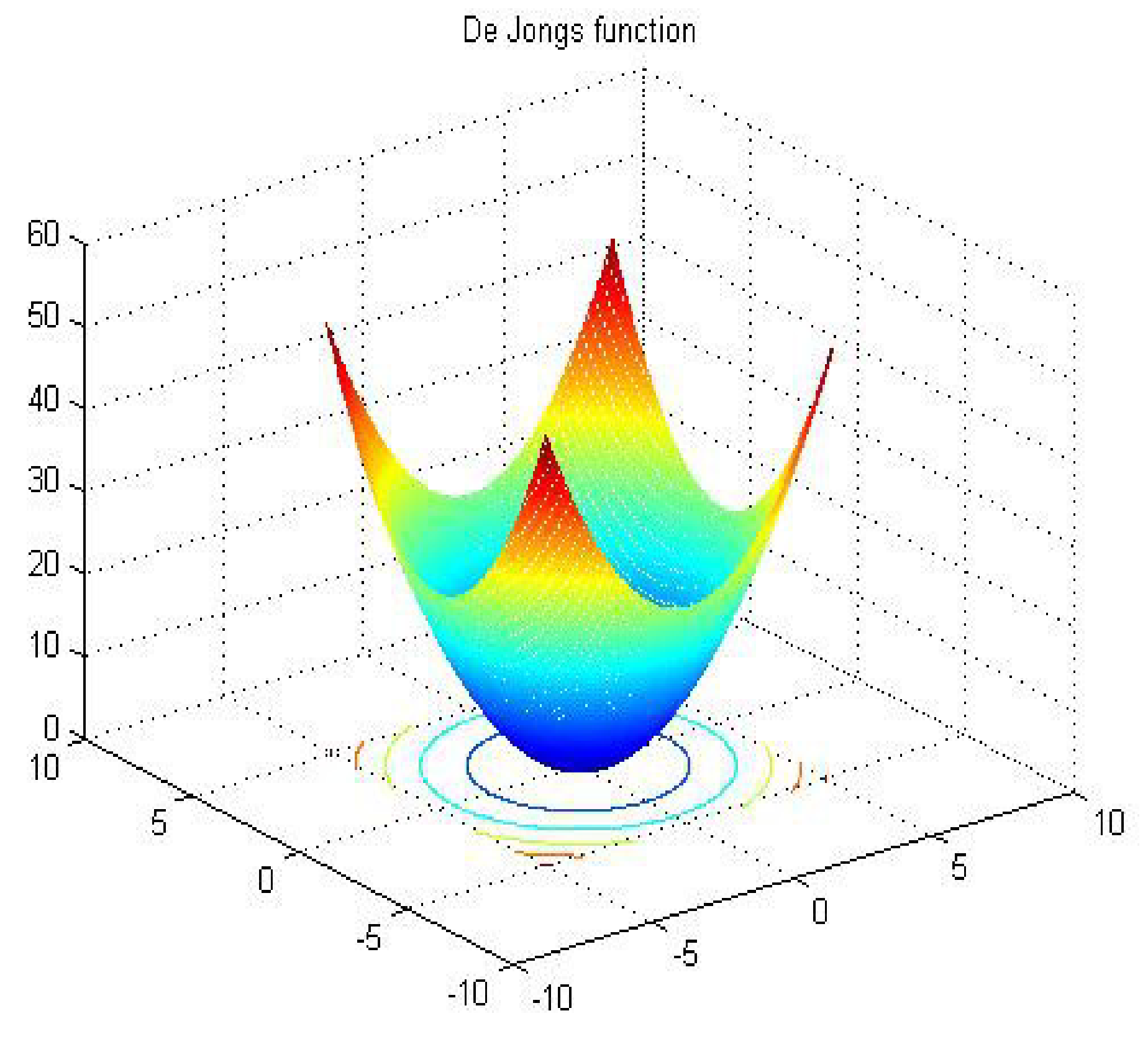

Supplement: S1 Appendix — (ZIP) [file pone.0144371.s001.zip › PONE-D-15-11851/Fig A.tiff]

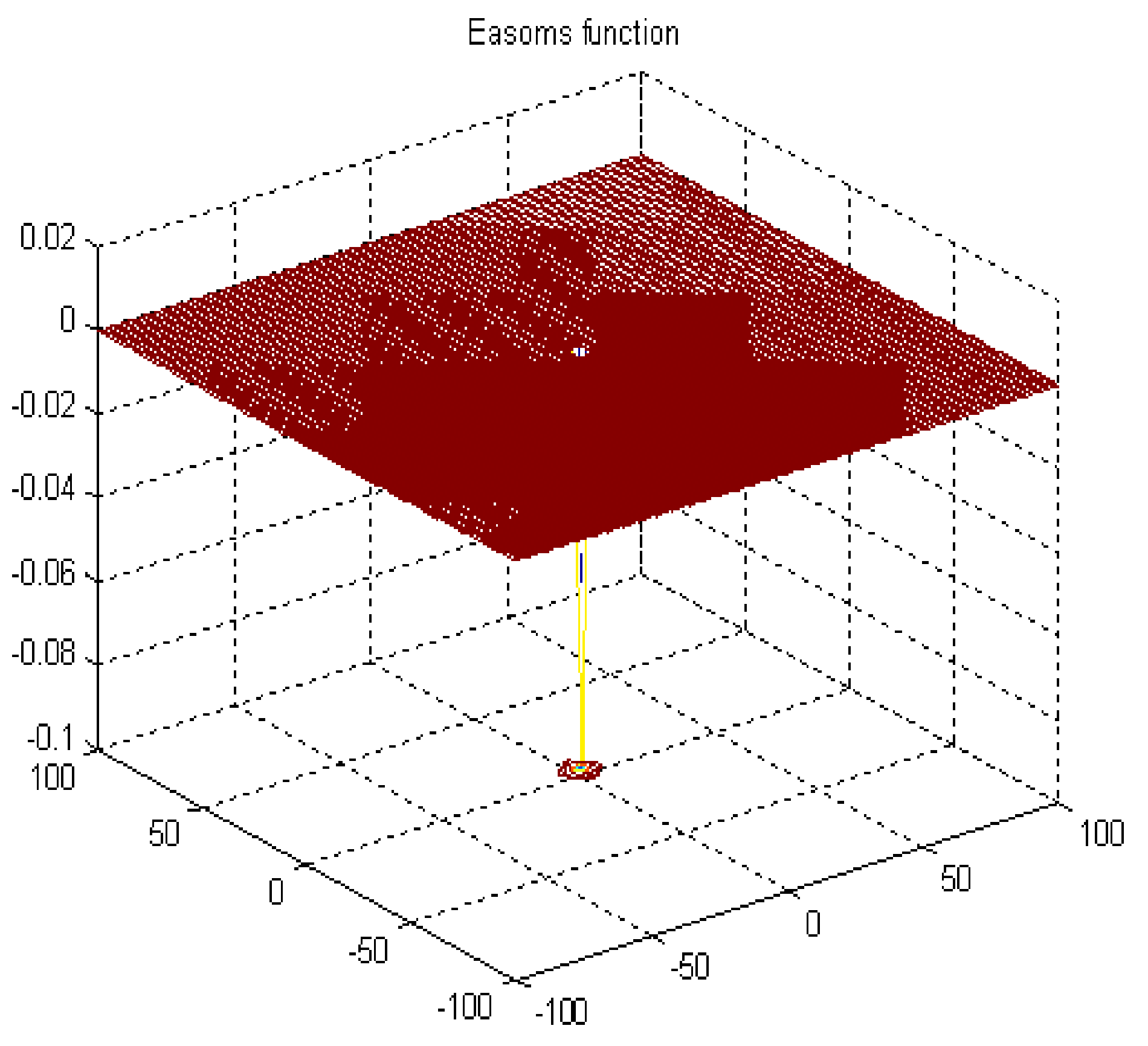

Supplement: S1 Appendix — (ZIP) [file pone.0144371.s001.zip › PONE-D-15-11851/Fig B.tiff]

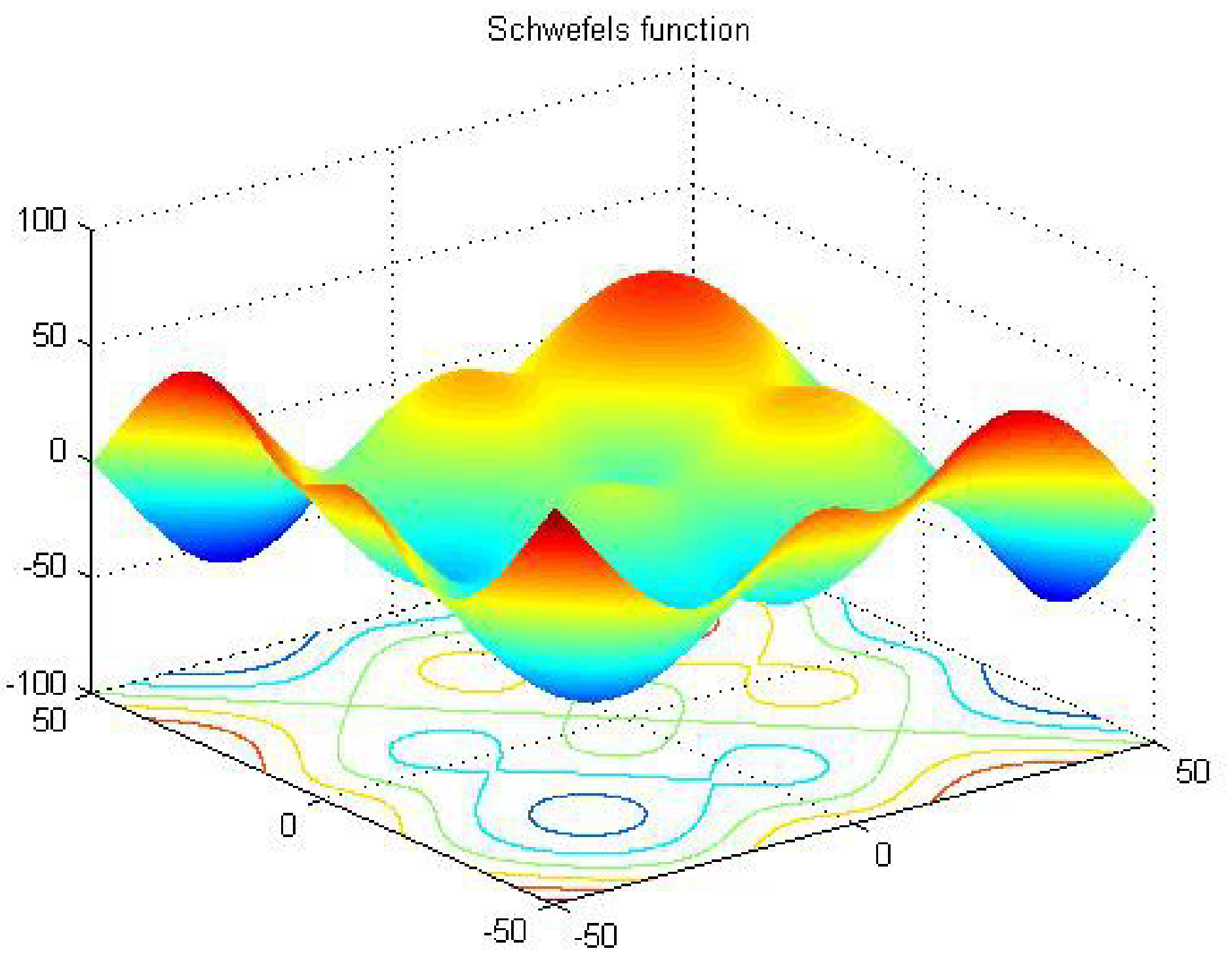

Supplement: S1 Appendix — (ZIP) [file pone.0144371.s001.zip › PONE-D-15-11851/Fig C.tiff]

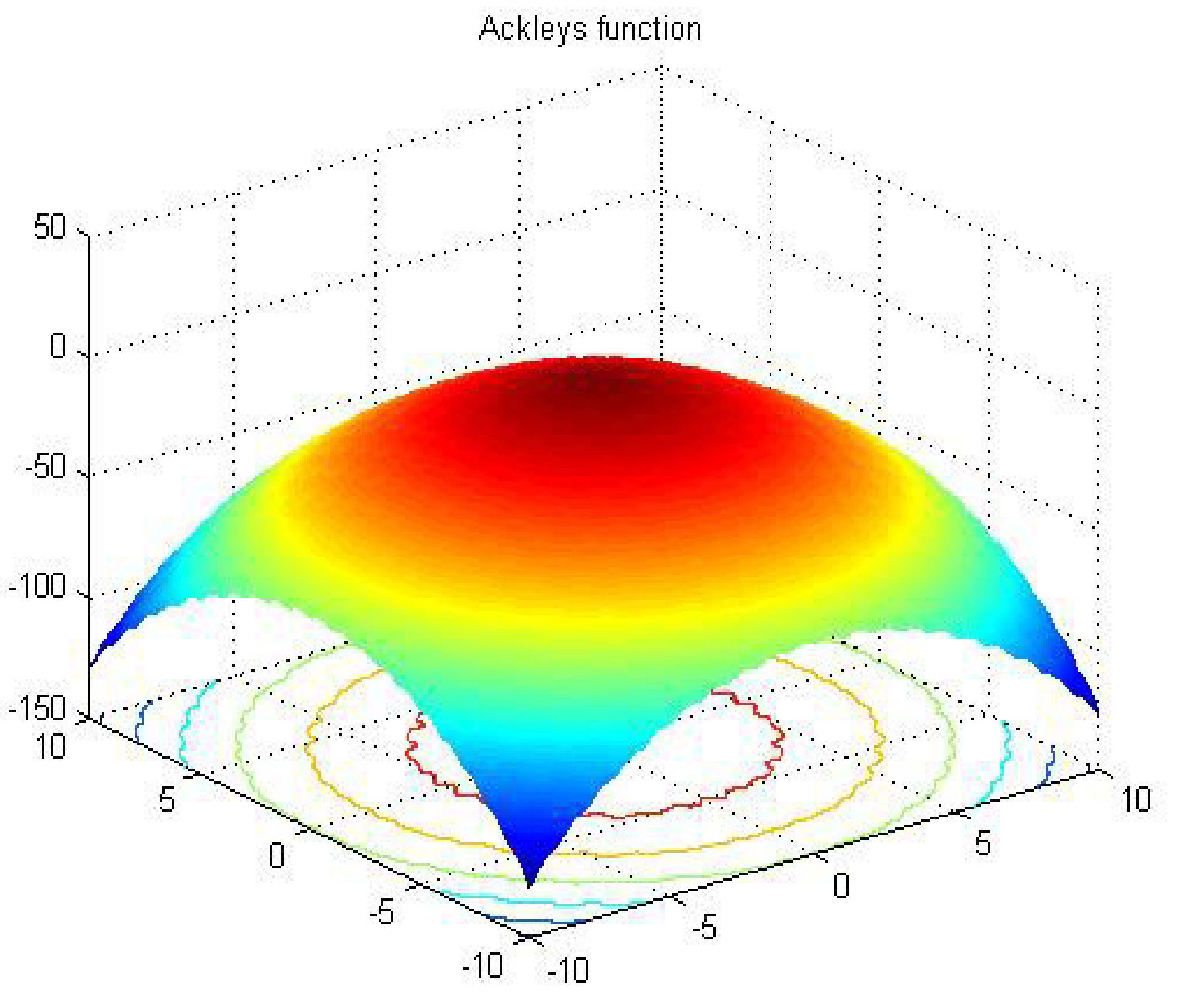

Supplement: S1 Appendix — (ZIP) [file pone.0144371.s001.zip › PONE-D-15-11851/Fig D.tiff]

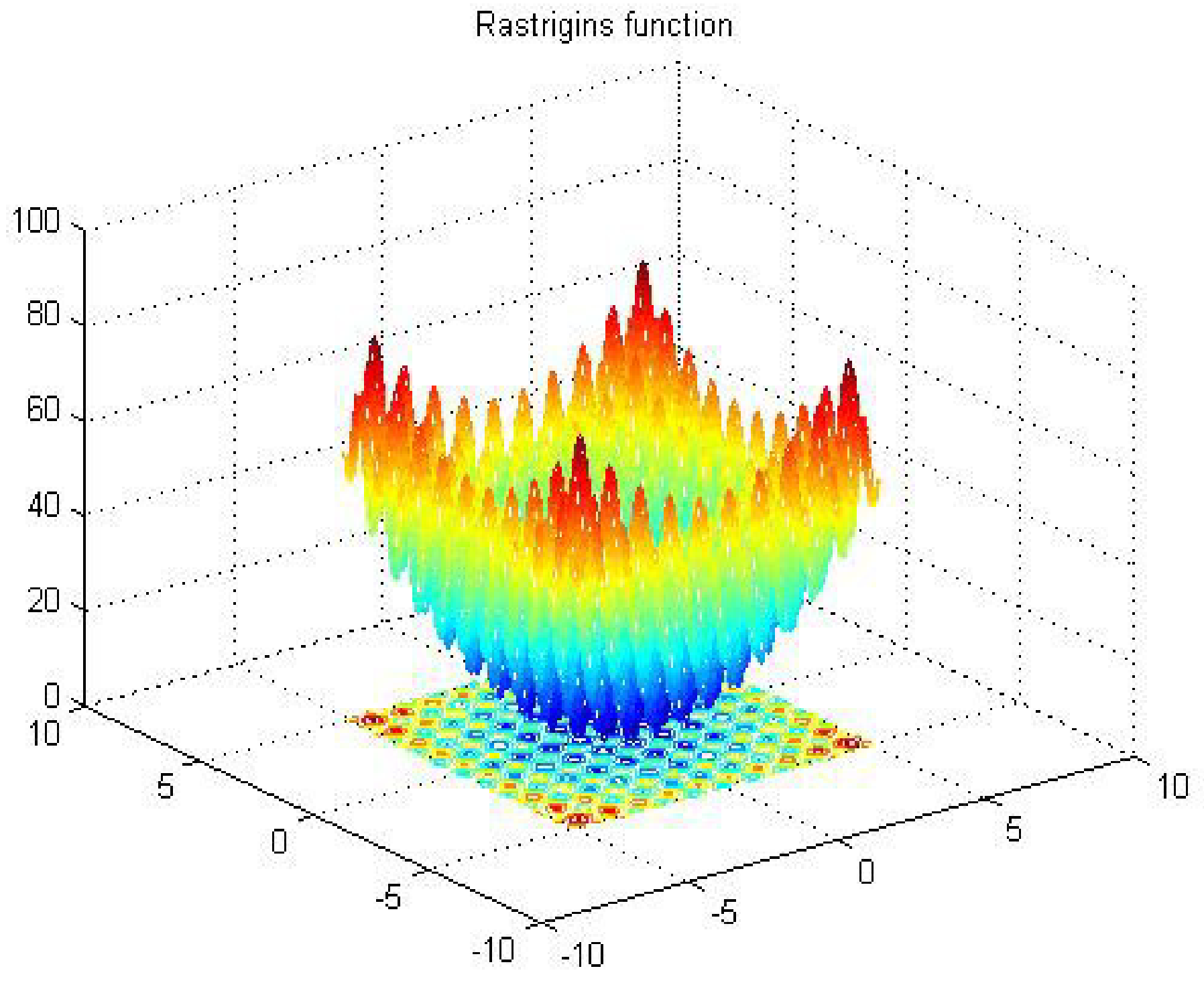

Supplement: S1 Appendix — (ZIP) [file pone.0144371.s001.zip › PONE-D-15-11851/Fig E.tiff]

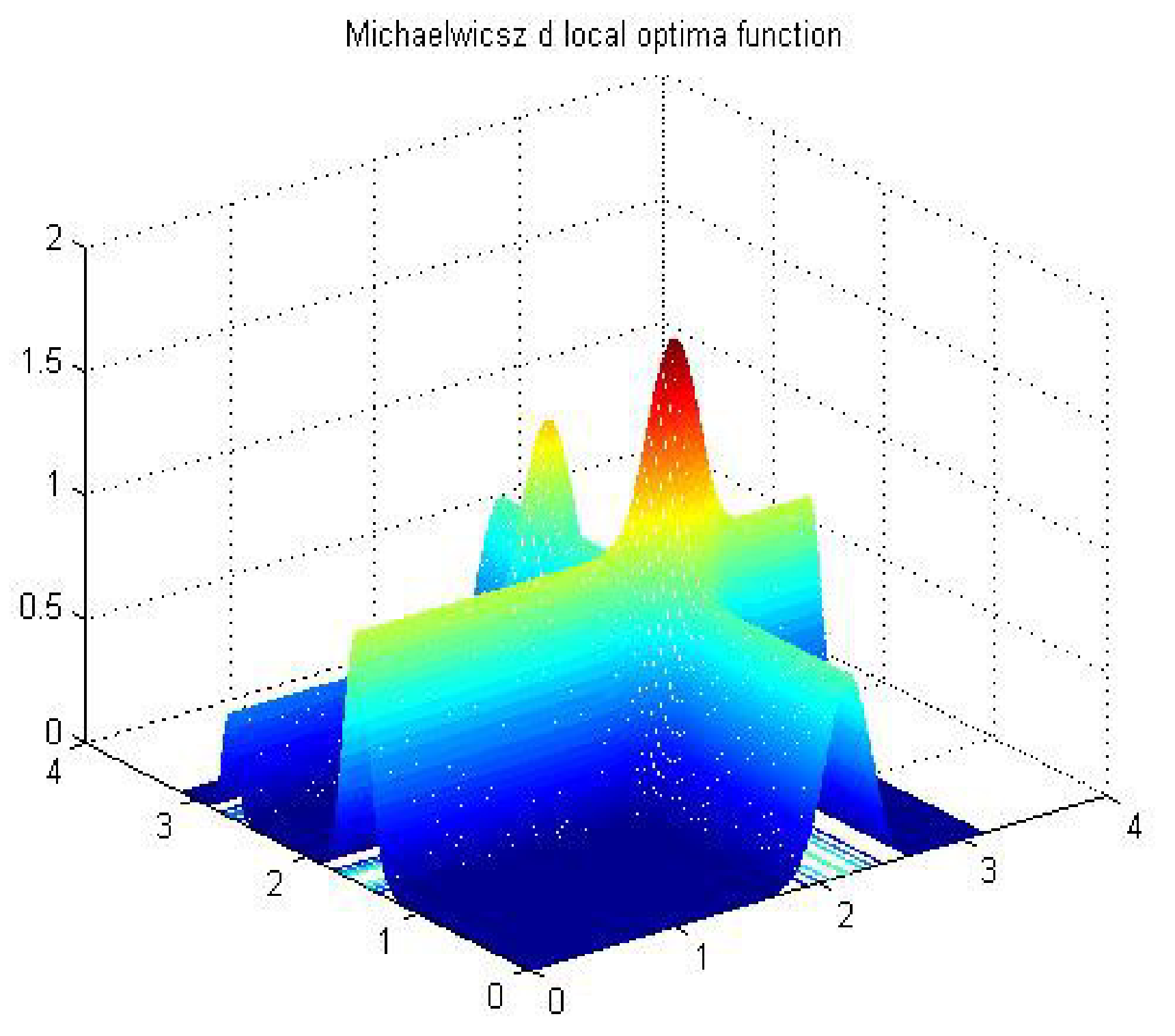

Supplement: S1 Appendix — (ZIP) [file pone.0144371.s001.zip › PONE-D-15-11851/Fig F.tiff]

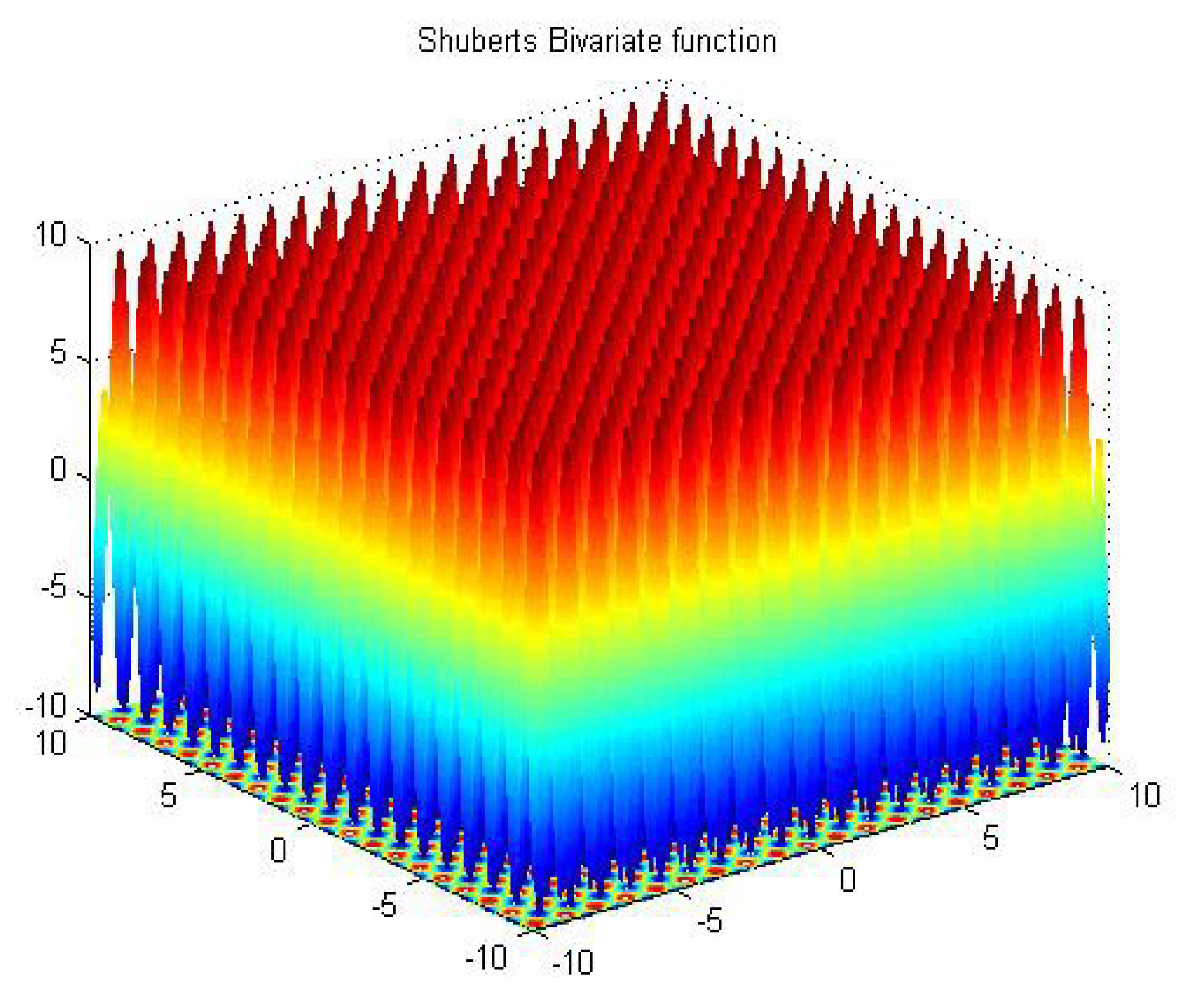

Supplement: S1 Appendix — (ZIP) [file pone.0144371.s001.zip › PONE-D-15-11851/Fig G.tiff]

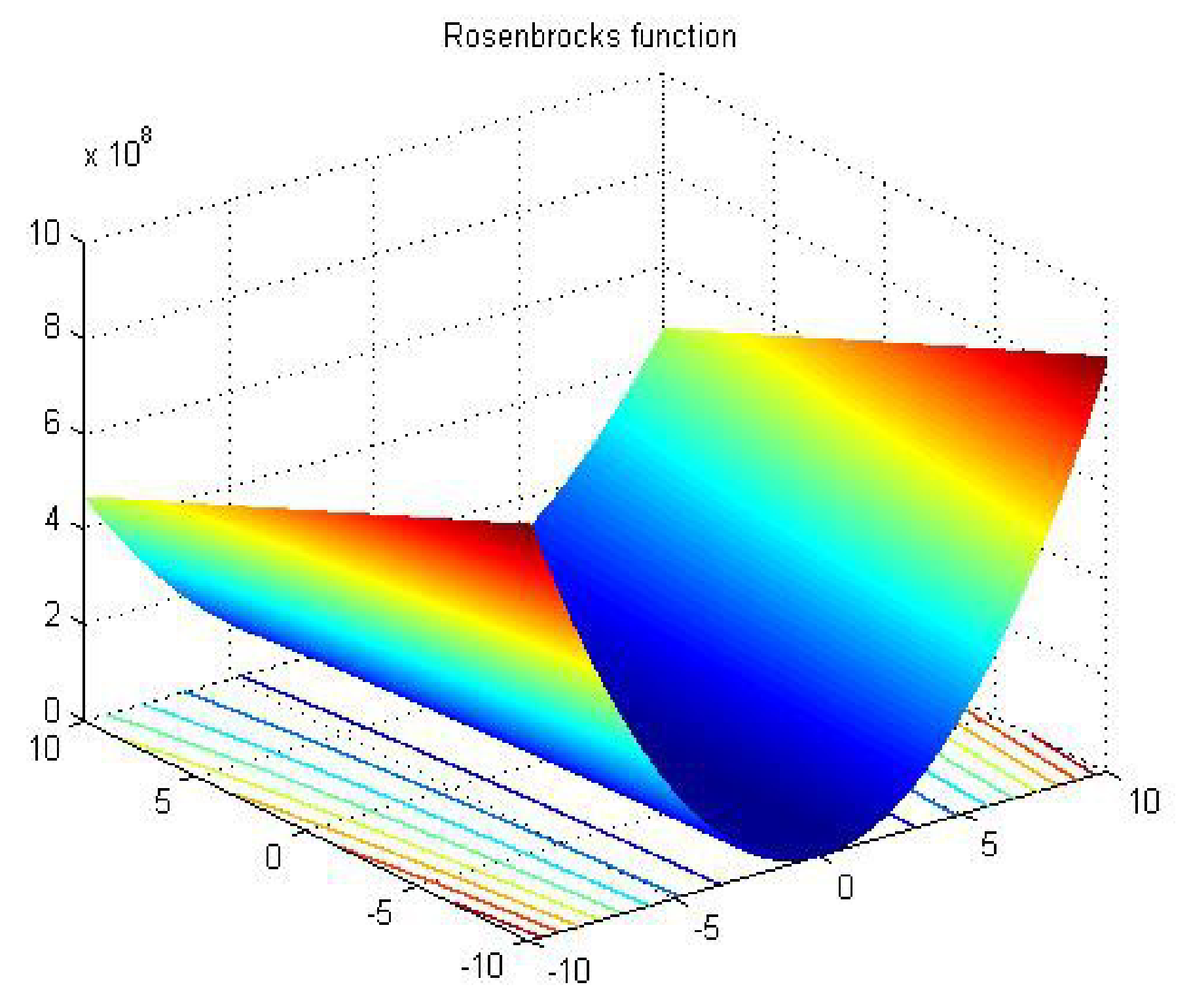

Supplement: S1 Appendix — (ZIP) [file pone.0144371.s001.zip › PONE-D-15-11851/Fig H.tiff]

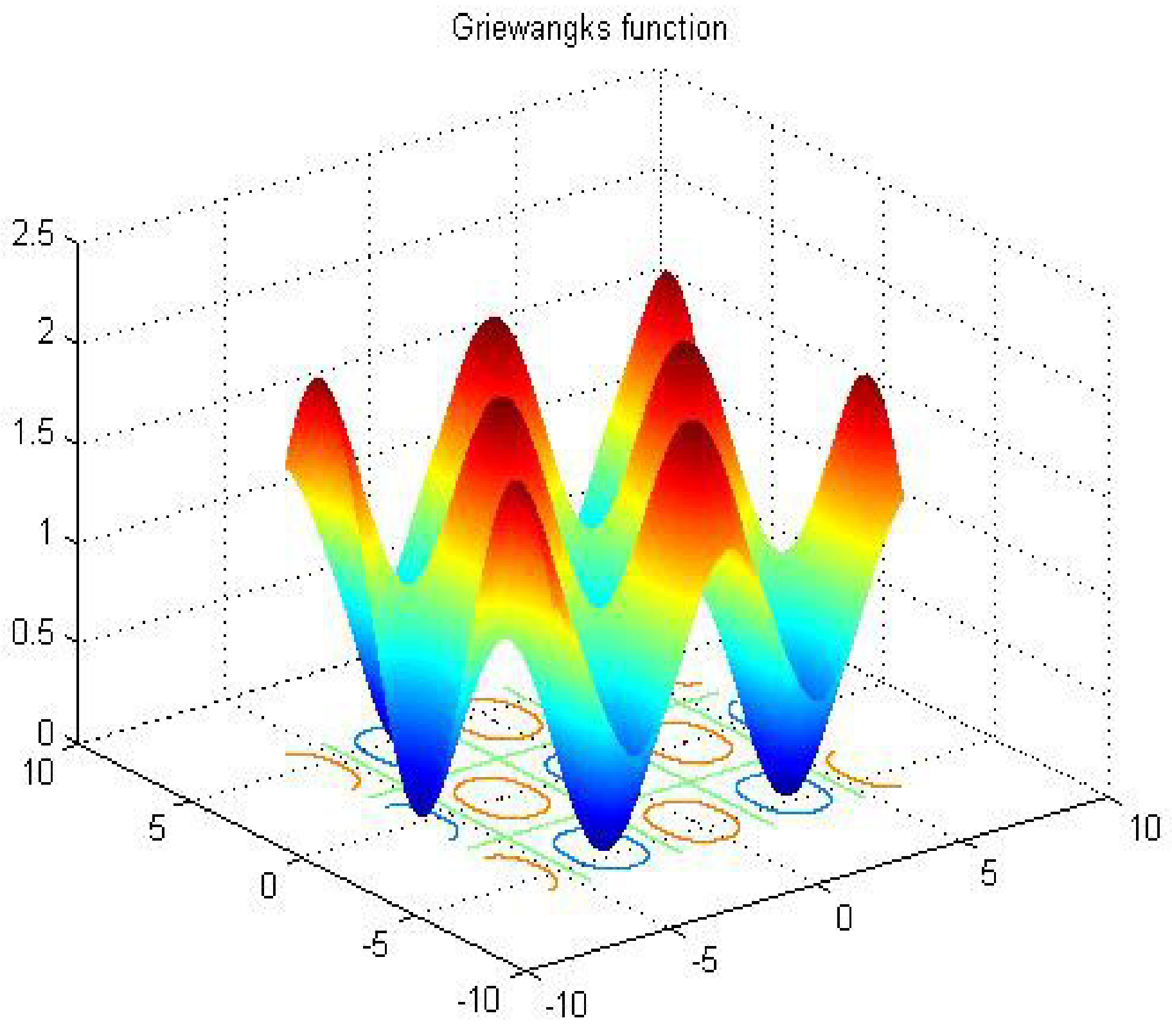

Supplement: S1 Appendix — (ZIP) [file pone.0144371.s001.zip › PONE-D-15-11851/Fig I.tiff]

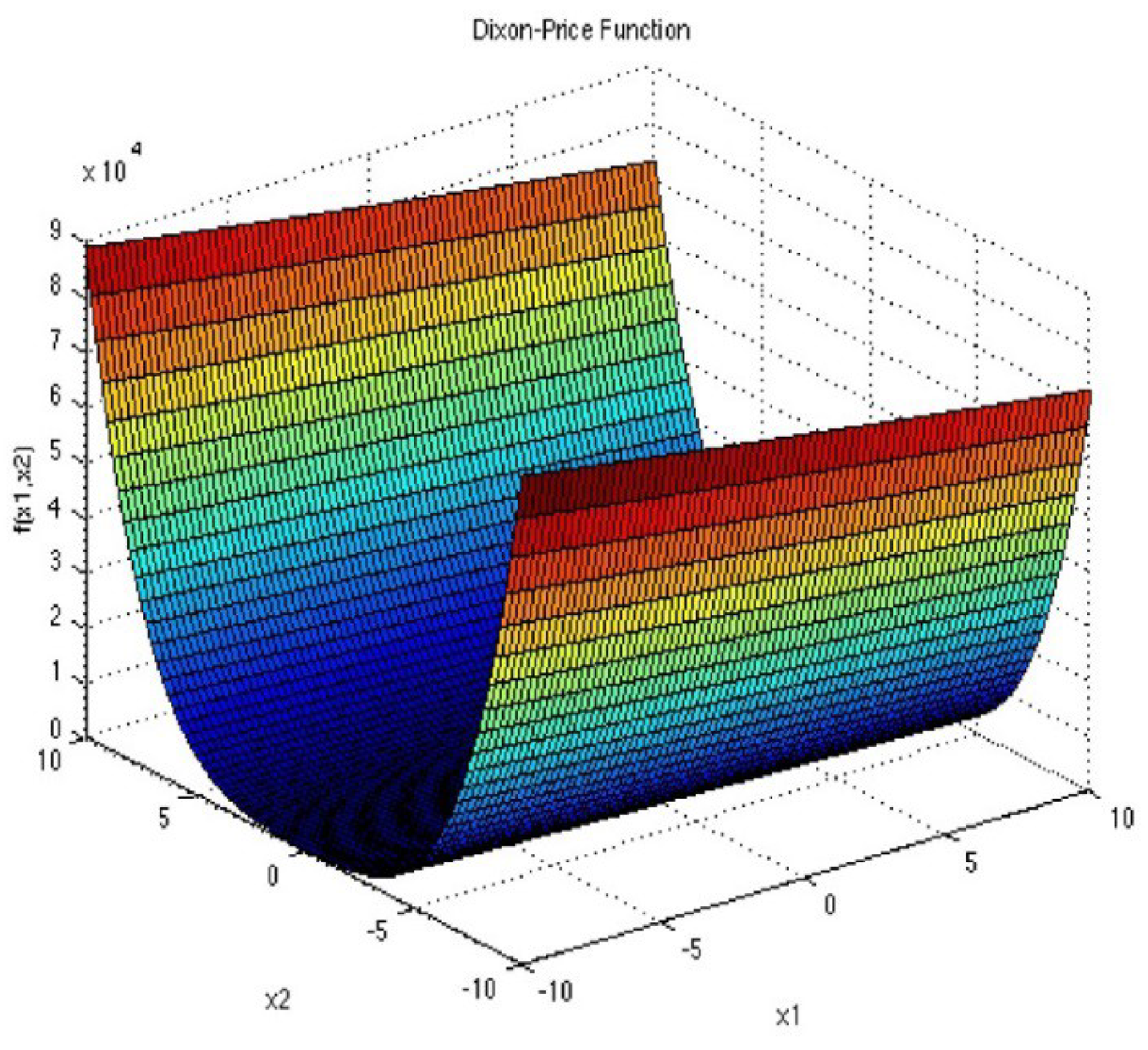

Supplement: S1 Appendix — (ZIP) [file pone.0144371.s001.zip › PONE-D-15-11851/Fig J.tiff]

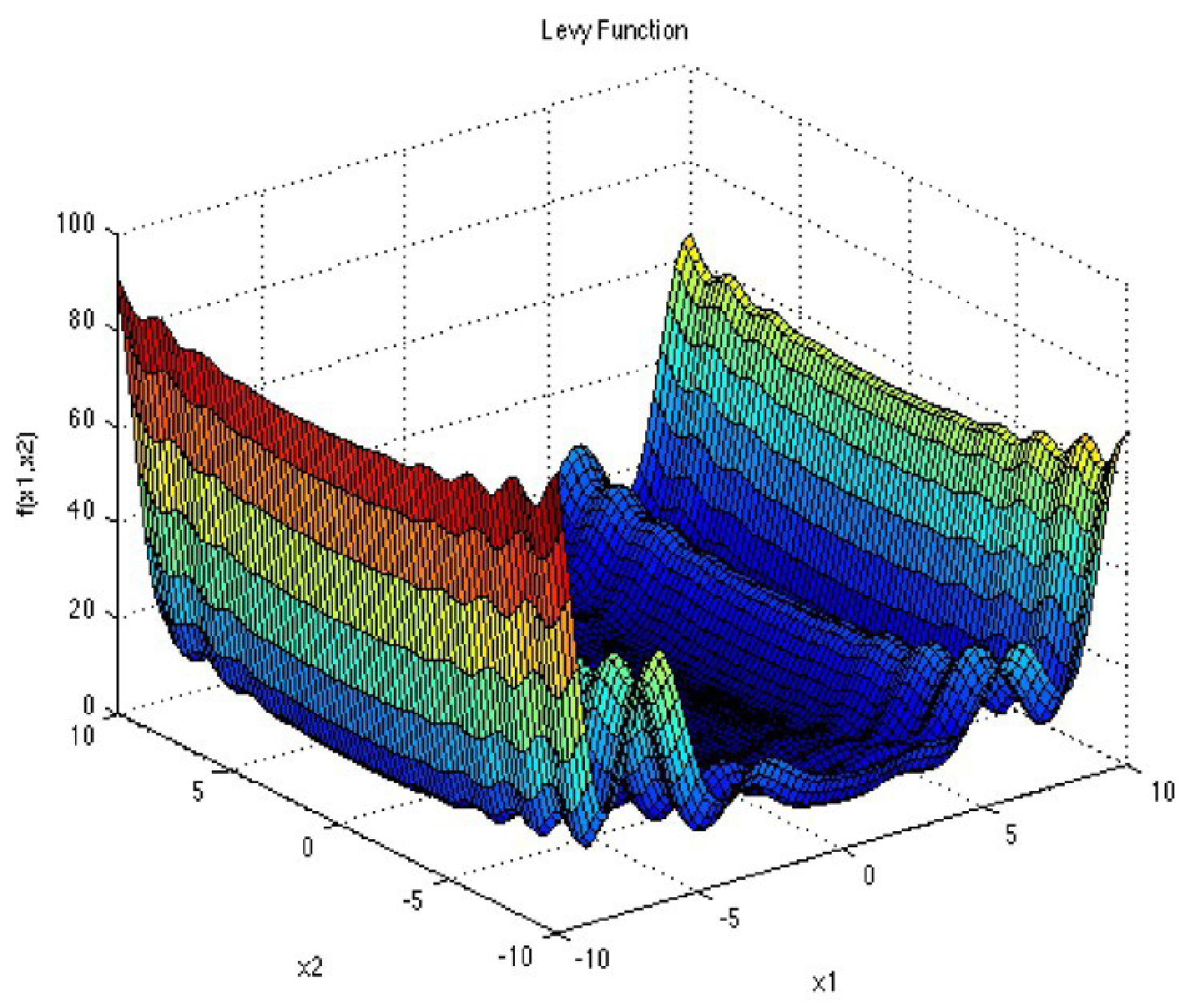

Supplement: S1 Appendix — (ZIP) [file pone.0144371.s001.zip › PONE-D-15-11851/Fig K.tiff]

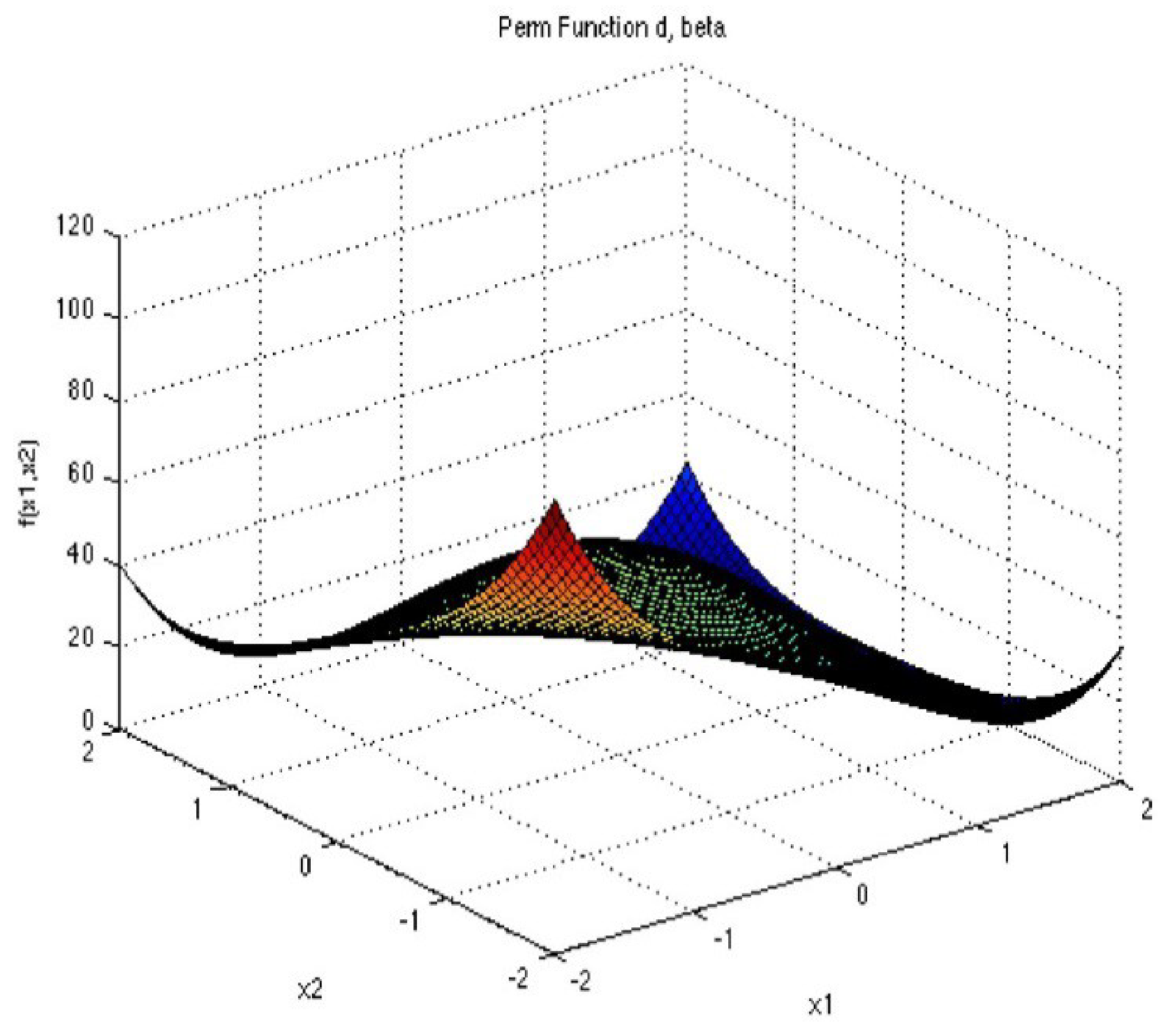

Supplement: S1 Appendix — (ZIP) [file pone.0144371.s001.zip › PONE-D-15-11851/Fig L.tiff]

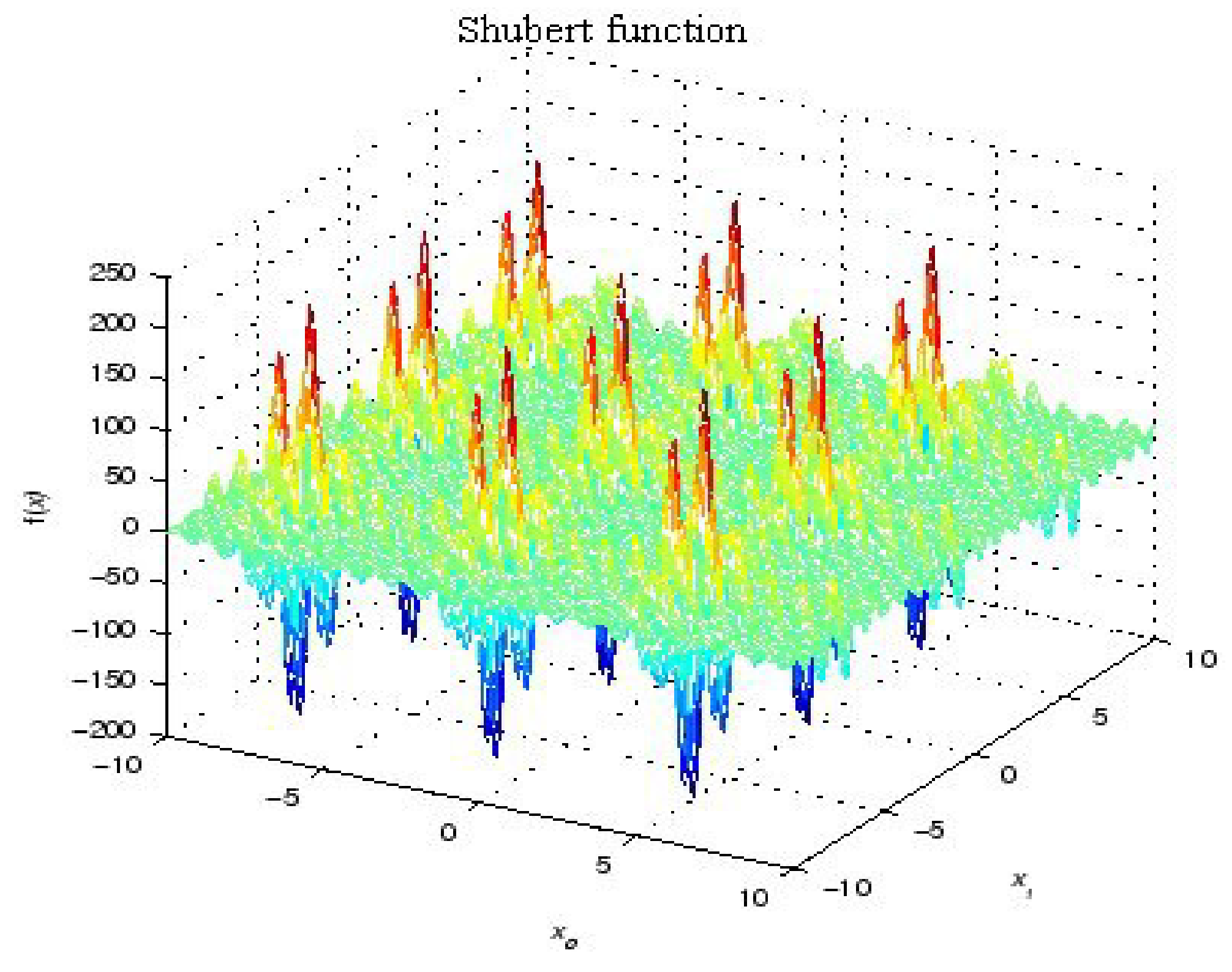

Supplement: S1 Appendix — (ZIP) [file pone.0144371.s001.zip › PONE-D-15-11851/Fig M.tiff]

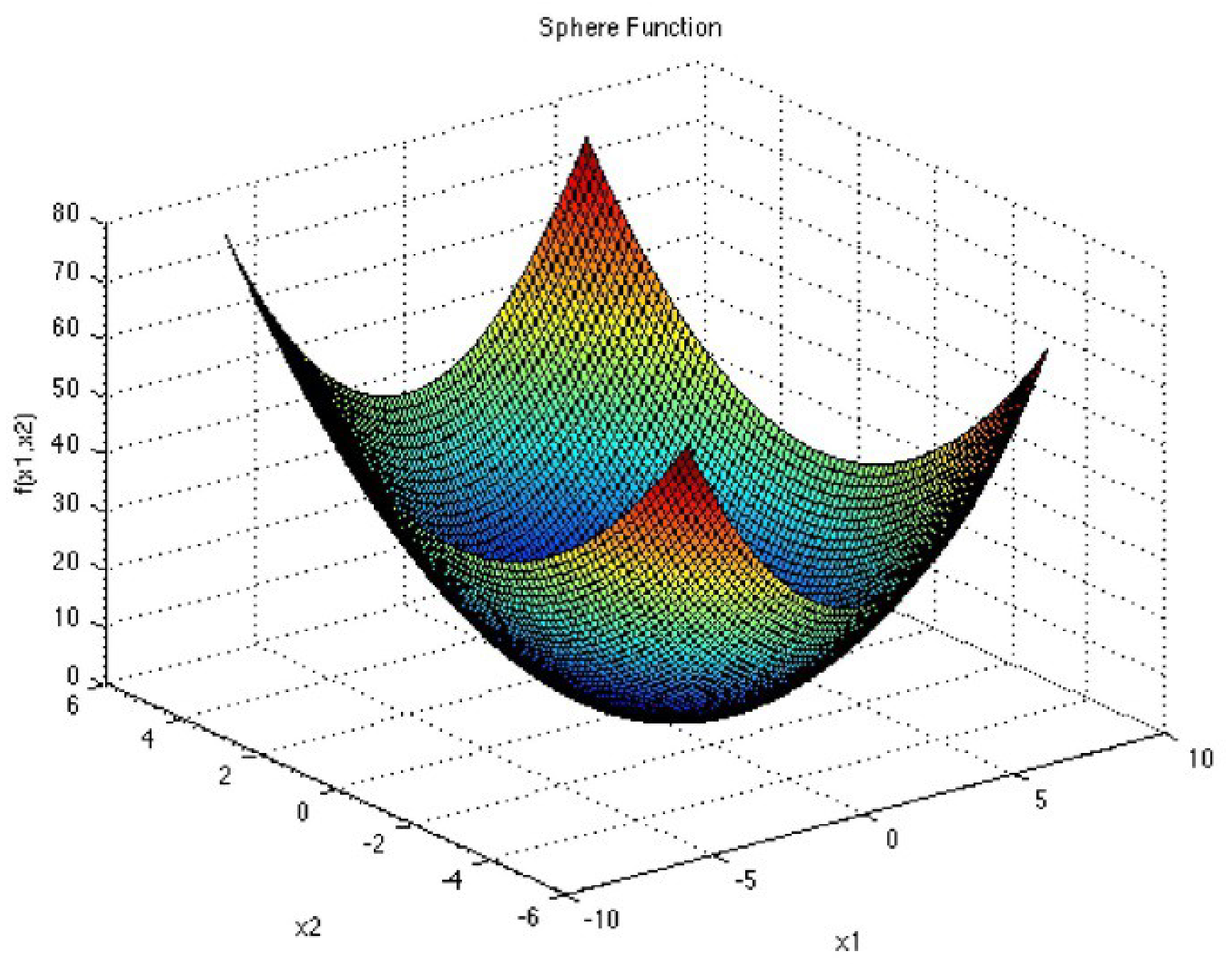

Supplement: S1 Appendix — (ZIP) [file pone.0144371.s001.zip › PONE-D-15-11851/Fig N.tiff]
