# Supplementary material for: Ringed Seal Search for Global Optimization via a Sensitive Search Model
Source: PLoS One. 2016 Jan 20;11(1):e0144371. doi: 10.1371/journal.pone.0144371 (PMC4720396; doi:10.1371/journal.pone.0144371)
Supplement: S1 Appendix — (ZIP) [file pone.0144371.s001.zip › PONE-D-15-11851/Appendix Fig Captions.doc]

**S1 Appendix: The landscaped of the test benchmark functions**

**Fig A. The landscaped of $F_{1}$ test function	Fig B. The landscaped of $F_{2}$ test function**

**Fig C. The landscaped of $F_{3}$ test function	Fig D. The landscaped of $F_{4}$ test function**

**Fig E. The landscaped of $F_{6}$ test function	Fig F. The landscaped of $F_{7}$ test function**

**Fig G. The landscaped of $F_{8}$ test function	Fig H. The landscaped of $F_{9}$ test function**

# Fig I. The landscaped of $F_{10}$ test function Fig J. The landscaped of $F_{11}$ test function

**Fig K. The landscaped of $F_{12}$ test function	Fig L. The landscaped of $F_{13}$ test function**

**Fig M. The landscaped of $F_{14}$ test function Fig N. The landscaped of $F_{15}$ test function**
